# SRSF1 inhibits autophagy through regulating Bcl-x splicing and interacting with PIK3C3 in lung cancer

**DOI:** 10.1038/s41392-021-00495-6

**Published:** 2021-03-05

**Authors:** Yuesheng Lv, Wenjing Zhang, Jinyao Zhao, Bing Sun, Yangfan Qi, Haoyu Ji, Chaoqun Chen, Jinrui Zhang, Junxiu Sheng, Taishu Wang, Daniel Dominguez, Han Liu, Quentin Liu, Songshu Meng, Xiaoling Li, Yang Wang

**Affiliations:** 1 Second Affiliated Hospital, Institute of Cancer Stem Cell, Dalian Medical University, Dalian, China; 2 Department of Thoracic Surgery, First Affiliated Hospital, Dalian Medical University, Dalian, China; 3 Department of Radiation Oncology, First Affiliated Hospital, Dalian Medical University, Dalian, China; 4 Department of Pharmacology, University of North Carolina at Chapel Hill, Chapel Hill, NC USA; 5 Signal Transduction Laboratory, National Institute of Environmental Health Sciences, Research Triangle Park, NC USA

**Keywords:** Cell biology, Lung cancer

## Abstract

Alternative splicing is a critical process to generate protein diversity. However, whether and how alternative splicing regulates autophagy remains largely elusive. Here we systematically identify the splicing factor SRSF1 as an autophagy suppressor. Specifically, SRSF1 inhibits autophagosome formation by reducing the accumulation of LC3-II and numbers of autophagosomes in different cell lines. Mechanistically, SRSF1 promotes the splicing of the long isoform of Bcl-x that interacts with Beclin1, thereby dissociating the Beclin1-PIK3C3 complex. In addition, SRSF1 also directly interacts with PIK3C3 to disrupt the interaction between Beclin1 and PIK3C3. Consequently, the decrease of SRSF1 stabilizes the Beclin1 and PIK3C3 complex and activates autophagy. Interestingly, SRSF1 can be degraded by starvation- and oxidative stresses-induced autophagy through interacting with LC3-II, whereas reduced SRSF1 further promotes autophagy. This positive feedback is critical to inhibiting Gefitinib-resistant cancer cell progression both in vitro and in vivo. Consistently, the expression level of SRSF1 is inversely correlated to LC3 level in clinical cancer samples. Our study not only provides mechanistic insights of alternative splicing in autophagy regulation but also discovers a new regulatory role of SRSF1 in tumorigenesis, thereby offering a novel avenue for potential cancer therapeutics.

## Introduction

Autophagy is a conserved intracellular degradation system among eukaryotes that functions to recycle redundant protein and malfunctioned organelles.^1^ Generally, two complexes are involved in the initiation stage of autophagy. One is the ULK1 complex (including ULK1, ULK2, ATG13, FIP200, and ATG101), and the other is the class III PI3K complex, which is composed of PIK3C3 (also known as VPS34), P150, and Beclin1.^2,3^ Beclin1, a protein that interacts with PIK3C3, is a crucial component in autophagy regulation and plays critical roles in tumorigenesis, development, and neurodegeneration.^4–6^

Autophagy has opposite and context-dependent functions in tumor progression that depends on various biological factors.^7,8^ Suppression of autophagy by FIP200 deletion inhibits mammary tumorigenesis.^9^ The mTOR signaling pathway regulates protein synthesis and autophagy to stimulate tumor growth.^10,11^ It has also been demonstrated that inhibition of autophagy could cooperate with dual inhibitors of mTOR/PI3K to stimulate cancer cell death.^12^ However, many other components involved in activating autophagy are tumor suppressors, e.g., Beclin1, UVRAG, PTEN.^13–15^ For instance, Beclin1 heterozygous disruption leads to elevated cellular proliferation and decreased autophagy in vivo.^16^ Additionally, suppression of Beclin1 sustains growth factor-stimulated AKT and ERK activation leading to increased breast cancer cell invasion.^17^ Therefore, mutation of Beclin1 or other autophagy-related genes could contribute to the initiation and development of human cancers, indicating that activation of autophagy can suppress tumorigenesis.

RNA alternative splicing plays an important role in regulating mRNA and protein diversity. The same pre-mRNA can generate different mature mRNAs by using various alternative splicing modes, thus to translate into distinct proteins with multiple functions.^18^ Remarkably, RNA splicing is tightly controlled in different tissues and developmental stages. Deregulated RNA splicing will lead to numerous human diseases, including cancer.^19,20^

Generally, alternative splicing can be regulated by a number of *cis*-elements, which recruit splicing factors to influence the usage of different splice sites.^21–23^ Dysregulation of alternative splicing can be led by mutations of *cis*-elements that abolish the utilization of the right splice sites. For example, a mutation at the splice site of BRCA1 abrogated nuclear localization and DNA response activities.^24^ On the other hand, the alteration of localization, activity, and expression level of splicing factors is thought to be another major cause of splicing dysregulation in cancer as well. For instance, the splicing factor SRSF6 is amplified in lung and colon cancers, which can regulate cancer-related RNA splicing to promote cancer progression.^25,26^ Similarly, SRSF1 is also identified as a proto-oncogene that regulates splicing of important cancer-related genes to promote tumorigenesis.^27–29^ However, the possible functions and molecular mechanisms of alternative splicing, particularly splicing factors, in autophagy regulation remain largely unknown.

Here we systematically identified splicing factors that play vital roles in autophagy regulation. We discovered that depleted SRSF1 activates autophagy. Mechanistically, SRSF1 regulates alternative splicing of Bcl-x towards the long isoform, thereby suppressing autophagy through interacting with Beclin1. Conversely, reduced level of SRSF1 promotes the production of Bcl-xS to abolish such interaction, thus to induce autophagy. In addition, SRSF1 directly interacts with PIK3C3 to disrupt the association of Beclin1 and PIK3C3, whereas knockdown of SRSF1 fails to dissociate the Beclin1 and PIK3C3 complex. Interestingly, the levels of SRSF1 are significantly decreased with the induction of starvation and oxidative stresses, suggesting that SRSF1 is degraded through autophagy. Most importantly, knockdown of SRSF1 inhibits Gefitinib-resistant cancer cell progression at least partially through activating autophagy. Taken together, our study highlights mechanistic insights of alternative splicing in autophagy, and provides a new regulatory role of SRSF1 in tumorigenesis, offering a novel avenue for potential cancer therapeutics.

## Results

### SRSF1 suppresses autophagy by inhibiting autophagosome formation

Activation of autophagy is an important approach to inhibit tumorigenesis. Several key regulators of autophagy, Beclin1 for example, have been shown to undergo alternative splicing to generate functionally distinct isoforms.^30^ However, splicing factors that regulate these alternative splicing events, thereby modulating autophagy and tumorigenesis, are still elusive.

**Fig. 1:**
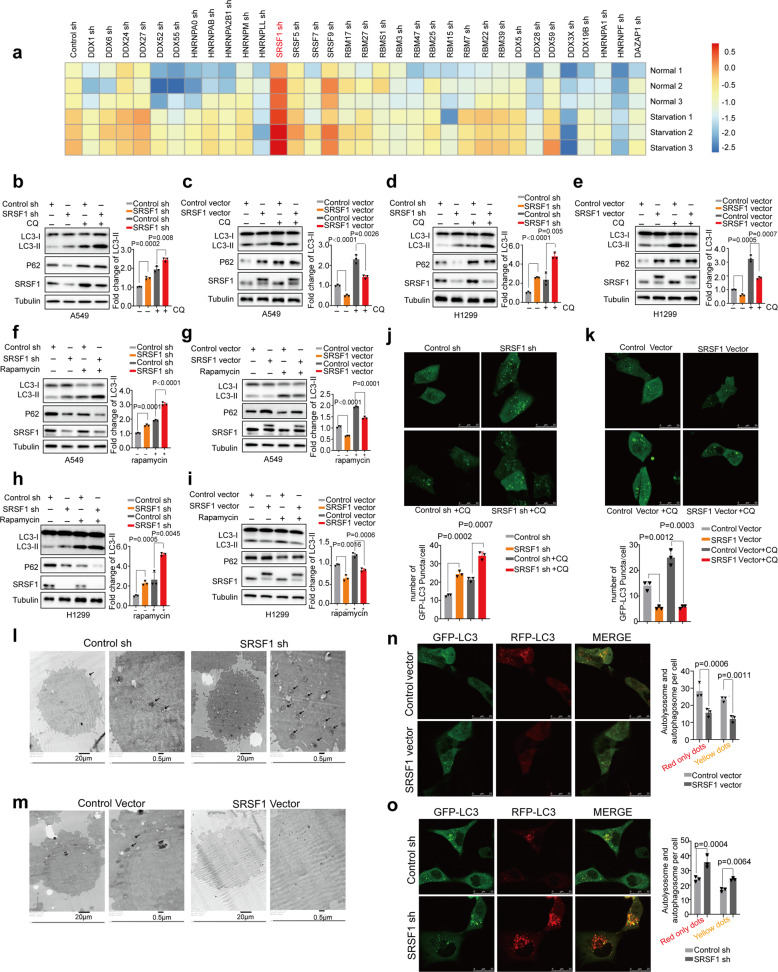
SRSF1 suppresses autophagy by inhibiting autophagosome formation. **a** Heatmap depicting the log2-scaled level of LC3-II/Tubulin when splicing factors were knocked down by distinct shRNAs in starved and control A549 lung cancer cells. Three replicates were shown for each group. **b**, **c** The protein levels of LC3, p62, and SRSF1 were examined in A549 cells with stable knockdown or overexpression of SRSF1 or control. Cells were treated without or with CQ (40 µM for 2 h). **d**, **e** The protein levels of LC3, p62, and SRSF1 were measured in H1299 cells with stable knockdown or overexpression of SRSF1 or control vector in the absence or presence of CQ (40 µM for 2 h). **f**, **g** A549 cells stably decreased or overexpressed SRSF1 or control were treated with Rapamycin, an inhibitor of mTOR pathway. The protein levels of LC3, p62, and SRSF1 were determined. **h**, **i** H1299 cells stably decreased or overexpressed SRSF1 or control were treated with Rapamycin, and the protein levels of LC3, p62, and SRSF1 were examined. The densities of signals were determined by densitometry and three experiments were carried out with mean ± SD of relative fold change of LC3-II plotted in **b**–**i**. **j** A549 cells with stable reduction of SRSF1 or control were transfected with GFP-LC3. Twenty-four hours later, cells were treated with or without CQ for 4 h. Three experiments were performed and the number of GFP-LC3 puncta per cell are represented with mean ± SD. **k** A549 cells stably overexpressed SRSF1 or control vector were transfected with GFP-LC3. Twenty-four hours later, cells were treated with or without CQ (40 µM) for 4 h. Three experiments were performed and the number of GFP-LC3 puncta per cell are represented with mean ± SD. **l**, **m** The autophagosomes of A549 cells with stable knockdown or overexpression of SRSF1 were examined with transmission electron microscopy (TEM). Autophagosomes was indicated by black arrows. **n**, **o** A549 cells with stable knockdown or overexpression of SRSF1 were transfected with GFP-mRFP-LC3 to examine the expression of GFP and RFP by confocal microscope. Three experiments were carried out with mean ± SD of the number of autophagosomes (yellow dots) and autolysosomes (red-only dots) per cell plotted. The *p* values were calculated by *t*-test in all panels

To systematically identify splicing factors that might participate in autophagy regulation in A549 lung cancer cells, we performed a screen with shRNAs, which have been verified by ENCODE project,^31^ to deplete several dozens of RNA binding proteins, respectively, and determined the regulation of autophagy as judged by the change of LC3-II level. Strikingly, we found that under both the normal and serum starvation conditions, as compared to control, SRSF1 was one of the top hits that substantially affected the accumulation of LC3-II, a marker of autophagic activity, suggesting that SRSF1 might play key roles in autophagy regulation (Fig. 1a, and Supplementary Fig. 1a, 1b). Further validation revealed that knockdown of SRSF1 indeed increased the accumulation of LC3-II, but decreased the expression of P62 in A549 and H358 lung cancer cells (Fig. 1b and Supplementary Fig. 1c). Conversely, overexpression of SRSF1 reduced the protein level of LC3-II, and elevated the expression of P62 (Fig. 1c and Supplementary Fig. 1d). Increased LC3-II indicates an accumulation of autophagosomes; however, such accumulation could result from either the induction of autophagy or reduced autophagosome turnover.^32^ To investigate which of these possibilities SRSF1 influences, we applied chloroquine (CQ) to interfere with the autophagosome-lysosome, an indicator of autophagic carrier flux.^32^ We treated cells with 40 µM CQ for 2 h, and found that knockdown of SRSF1 could still promote LC3-II level, whereas SRSF1 overexpression inhibited LC3-II level, indicating that SRSF1 could influence the autophagosome formation (Fig. 1b, c, and Supplementary Fig. 1c, d).

Since it has been shown that SRSF1 could stabilize p53,^33^ a known regulator of autophagosome formation through the canonical AMPK-mTOR signaling pathway. we thus used H1299 lung cancer cells that lack expression of p53 protein, to examine whether SRSF1 influences autophagy through p53 pathway. Importantly, we found that SRSF1 still affected autophagy in H1299 cells in the absence or presence of CQ (40 µM for 2 h), implying that such autophagy regulation is dominated by p53-independent pathway (Fig. 1d, e). In addition, we found that re-expression of SRSF1 could suppress the SRSF1-depletion-induced autophagy in both A549 and H1299 cells (Supplementary Fig. 1e). Previously, SRSF1 has been demonstrated to play vital roles in mTOR pathway that is closely linked to autophagic activation. We therefore treated cells with Rapamycin, an mTOR pathway inhibitor, to examine whether SRSF1 influences autophagy through the mTOR pathway. We found that SRSF1 could impact autophagy after the mTOR pathway was blocked (Fig. 1f–i, and Supplementary Fig. 1f, g). Importantly, SRSF1 was further revealed to suppress the accumulation of LC3-II in the context of hydrogen peroxide (H_2_O_2_) and sodium arsenite treatment both in the absence and presence of CQ (40 µM for 2 h) (Supplementary Fig. 1h), suggesting that SRSF1 could affect the autophagosome formation step induced by different conditions. To rule out the possibility of off-target effect of downregulating SRSF1 with shRNAs, we also applied a panel of siRNAs to deplete SRSF1, and found that depletion of SRSF1 with siRNAs could activate autophagy in H1299 cells as well (Supplementary Fig. 1i). Additionally, we observed similar effect of SRSF1 on autophagy regulation in 293 T and HeLa cells in the absence and presence of CQ (40 µM for 2 h), indicating that SRSF1-regulated autophagy is not in a cell type dependent manner (Supplementary Fig. 1j–m).

We further analyzed the numbers of autophagosomes by confocal microscopy assay. Noticeably, downregulation of SRSF1 increased autophagic puncta, whereas overexpression of SRSF1 suppressed autophagic puncta in both A549 and HeLa cells (Fig. 1j, k, Supplementary Fig. 1n, o). These results were also validated by transmission electron microscopy (Fig. 1l, m, Supplementary Fig. 1p, q), suggesting that SRSF1 levels modulate autophagic activity across multiple cell lines. To further corroborate that SRSF1 mainly influences the autophagosome formation, we directly analyzed the autophagic flux by transfecting cells with a tandem mRFP-GFP-LC3 reporter (tfLC3) into cells, which will emit both RFP and GFP signals when targeted into autophagosomes but will only emit RFP signal in autolysosomes because of quenching of the GFP in the acidic lysosomal environment.^34^ As shown in Fig. 1n, o, Supplementary Fig. 1r, s, reduction of SRSF1 dramatically increased both the yellow dots (autophagosomes) and red-only dots (autolysosome), whereas overexpression of SRSF1 significantly decreased the number of both yellow and red-only vesicles, further proving that SRSF1 mostly affects the autophagosome formation. Together, our results indicate that reduced SRSF1 is associated with increased autophagosome formation and autophagy.

### SRSF1 controls Bcl-x splicing by directly binding to the pre-mRNAs

**Fig. 2:**
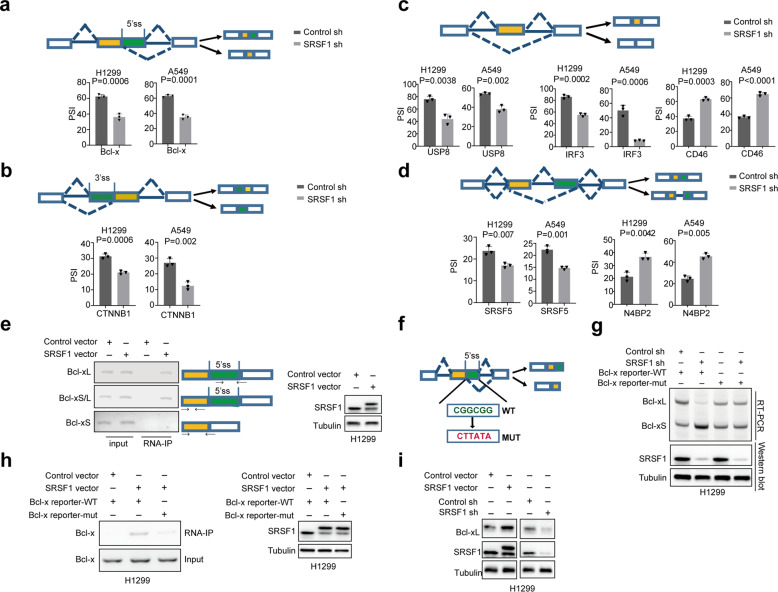
SRSF1 controls Bcl-x splicing by directly binding to the pre-mRNAs. **a** Alternative 5′ splice sites usage of Bcl-x was examined in H1299 and A549 cells with stably reduced SRSF1 or control. **b** Alternative 3′ splice sites usage of CTNNB1 was determined in H1299 and A549 cells with stable knockdown of SRSF1 or control. **c** Exon skipping in USP8, IRF3, and CD46 were measured in H1299 and A549 cells with stable decrease of SRSF1 or control. **d** Intron retention in SRSF5, and N4BP2 were investigated in H1299 and A549 cells with stable reduction of SRSF1 or control. The mean ± SD of PSIs from three experiments were plotted, *p* values were calculated by paired Student’s *t*-test in **a**–**d**. **e** Binding of Bcl-x pre-mRNAs with SRSF1 was examined by RNA-immunoprecipitation assay in H1299 cells exogenously expressed FLAG-SRSF1 or control vector. Three pairs primers were used to examine Bcl-xL, Bcl-xS/L, or Bcl-xS, respectively as shown. The protein levels of SRSF1 were measured with western blot. **f** The schematic of Bcl-x splicing reporters with the predicted SRSF1-binding site in green and the mutated site in red. **g** Bcl-x splicing reporters containing wild-type or mutated potential SRSF1-binding site were expressed in H1299 cells with stable knockdown of SRSF or control to examine the splicing change of Bcl-x. The protein levels of SRSF1 were also measured. Representative gel and blots were shown. **h** H1299 cells were co-transfected with Flag-SRSF1 or control vector and the wild-type or mutant Bcl-x reporters, and subsequently immunoprecipitated with anti-Flag antibody. The co-precipitated RNAs were utilized to examine the level of Bcl-x by RT-PCR. A representative gel was demonstrated. The protein levels of SRSF1 were examined with western blot. **i** The protein levels of Bcl-xL, and SRSF1 were examined in H1299 cells with stable overexpression of SRSF or control vector and stable reduction of SRSF1 or control in a western blot assay

To better understand the molecular mechanisms underlying SRSF1-regulated autophagy, we analyzed our previous RNA-seq data (GSE107224) that were obtained from cells stably knocked down SRSF1 or control. We found that SRSF1 could regulate alternative splicing of a number of autophagy-related genes, including the alternative usage of 5′ splice sites of Bcl-x, and 3′ splice sites of CTNNB1 (Fig. 2a, b, Supplementary Fig. 2a, b), as well as the exon skipping of USP8, IRF3, and CD46 (Fig. 2c, Supplementary Fig. 2a). Additionally, the intron retention of SRSF5, and N4BP2 was also modulated by SRSF1 (Fig. 2d, Supplementary Fig. 2a). Such splicing regulation was validated in both H1299 and A549 cells.

We specifically focused on the splicing regulation of Bcl-x, which is a key player involved in autophagy process.^35^ It has been reported that Bcl-x is a known target of SRSF1,^36–38^ but the detailed regulatory mechanisms are still unknown. We applied the online bioinformatics analyses (ESEfinder), and identified the potential SRSF1-binding site (CGGCGG sequence), which is similar to the previously reported SRSF1-binding motif,^39^ between two alternative 5′ splice sites within exon 2 of Bcl-x (Supplementary Fig. 2c), implying that SRSF1 might modulate the splicing of Bcl-x through directly binding to the pre-mRNA. In support of this possibility, SRSF1 bound to the endogenous Bcl-x pre-mRNAs in an RNA-IP assay in both H1299 and 293 T cells (Fig. 2e and Supplementary Fig. 2d). Most importantly, SRSF1 specifically bound to Bcl-xL, but not Bcl-xS, in the RNA-IP experiment as examined by the isoform-specific primers (Fig. 2e). Moreover, SRSF1 controlled the splicing of a transiently transfected exogenic mini-gene reporter containing the wild-type SRSF1-binding site of Bcl-x, but mutation of this binding site in the transiently transfected Bcl-x reporter abolished such splicing regulation by SRSF1 (Fig. 2f, g). Further RNA-IP assays with wild-type or mutated reporters confirmed our findings that the SRSF1-binding is indeed dependent on the CGGCGG site, as the mutation of this site almost completely prevented the interaction of SRSF1 with the Bcl-x pre-mRNA in both H1299 and 293 T cells (Fig. 2h and Supplementary Fig. 2e). This splicing shift led by SRSF1 also resulted in a robust change of Bcl-xL protein as judged by western blot (Fig. 2i and Supplementary Fig. 2f). Taken together, our data demonstrate that SRSF1 directly binds to the region between two 5′ splice sites of Bcl-x to promote the production of Bcl-xL isoform.

### Bcl-x isoforms differentially regulate autophagy

**Fig. 3:**
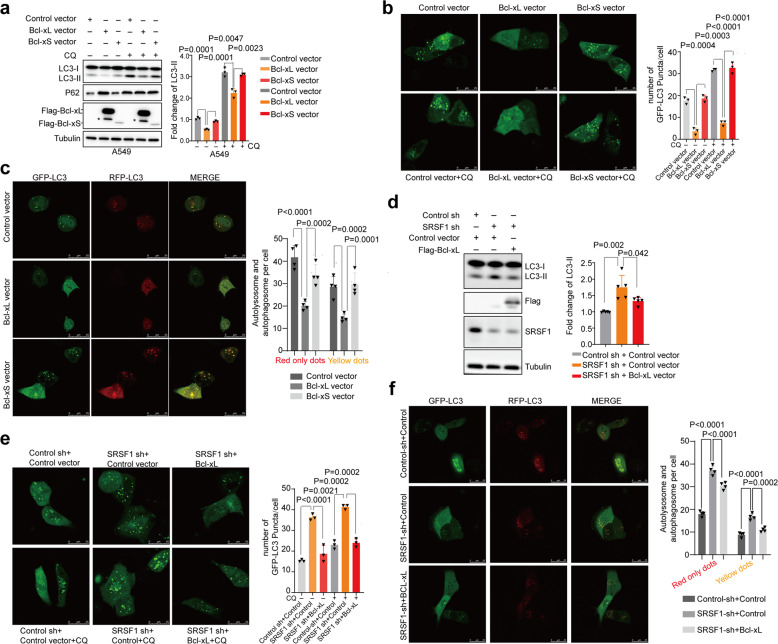
Bcl-x isoforms differentially regulate autophagy. **a** The protein levels of LC3 and p62 were determined in A549 cells with stable overexpression of Flag-Bcl-xL, Flag-Bcl-xS, or control vector. The resulting cells were treated without or with CQ (40 µM for 2 h) respectively. The densities of signals were measured by densitometry and three experiments were performed with mean ± SD of relative fold change of LC3-II plotted. Asterisk indicated nonspecific bands. **b** A549 cells with stable overexpression of Bcl-xL, Bcl-xS, or control vector were transfected with GFP-LC3 respectively. Twenty-four hours after transfection, cells were treated with or without CQ. Three experiments were carried out and the number of GFP-LC3 puncta per cell are represented with mean ± SD. **c** A549 cells with stably overexpressed Bcl-xL, Bcl-xS, or control vector were transfected with GFP-mRFP-LC3 to examine the expression of GFP and RFP by confocal microscope. Four experiments were carried out with mean ± SD of the number of autophagosomes (yellow dots) and autolysosomes (red-only dots) per cell plotted. **d** A549 cells with stable decrease of SRSF1 or control were transiently transfected with Flag-Bcl-xL or control vector. The protein levels of LC3 and SRSF1 were examined in a western blot assay. The densities of signals were analyzed by densitometry and five experiments were carried out with mean ± SD of relative fold change of LC3-II plotted. **e** A549 cells with stable decrease of SRSF1 or control were transiently transfected with Flag-Bcl-xL or control vector and GFP-LC3. Twenty-four hours after transfection, cells were treated with or without CQ. Three experiments were performed and the number of GFP-LC3 puncta per cell are represented with mean ± SD. **f** A549 cells with stable reduction of SRSF1 or control were transiently transfected with Flag-Bcl-xL or control vector and GFP-mRFP-LC3 to examine the expression of GFP and RFP by confocal microscope. Four experiments were carried out with mean ± SD of the number of autophagosomes (yellow dots) and autolysosomes (red-only dots) per cell plotted. The *p* values were calculated by *t*-test in all panels

We next sought to investigate whether both of the two splicing isoforms of Bcl-x participate in autophagy regulation. To this end, we stably overexpressed Bcl-xL or Bcl-xS in A549 cells, respectively. As reported previously, Bcl-xL markedly decreased the accumulation of LC3-II.^40^ However, we discovered that the short isoform, Bcl-xS, did not affect the amount of LC3-II and P62 as judged by a western blot assay (Fig. 3a). In addition, we treated cells with CQ to further assess the functions of Bcl-xL and Bcl-xS in autophagy regulation, and obtained similar results that only Bcl-xL influences the autophagy flux (Fig. 3a). Consistently, the number of autophagosomes was significantly reduced by Bcl-xL, but not by Bcl-xS, as examined by confocal microscopy assay in A549 and HeLa cells in the absence or presence of CQ (Fig. 3b, Supplementary Fig. 3a). Additional dual fluorescence assay for autophagy flux revealed that overexpression of Bcl-xL dramatically decreased the yellow dots (autophagosomes) and red-only dots (autolysosome), while Bcl-xS had no influence on the number of yellow and red-only vesicles (Fig. 3c, Supplementary Fig. 3b), suggesting that Bcl-xL, but not Bcl-xS, is involved in the inhibition of autophagosome formation at an early stage.

We further investigated whether the splicing switch of Bcl-x is responsible for the SRSF1-mediated autophagy regulation. To this end, we examined if re-expression of Bcl-xL can overturn the autophagy activation induced by SRSF1 downregulation. As shown in Fig. 3d, stable overexpression of Bcl-xL in A549 cells partially decreased SRSF1-reduction induced LC3-II accumulation. Conversely, knockdown of Bcl-xL in SRSF1 overexpressing cells could reverse the SRSF1-induced suppression of autophagy as judged by the accumulation of LC3-II (Supplementary Fig. 3c). Moreover, SRSF1-depletion-induced increase of autophagic puncta (Fig. 3e) and an increase of the yellow dots (autophagosomes) and red-only dots (autolysosome) (Fig. 3f) were also partially reduced by Bcl-xL expression. Collectively, these data demonstrate that SRSF1 inhibits autophagy at least in part through promoting the production of Bcl-xL.

### SRSF1-mediated splicing of Bcl-xL inhibits autophagy through disruption of the formation of the autophagy initiation complex

**Fig. 4:**
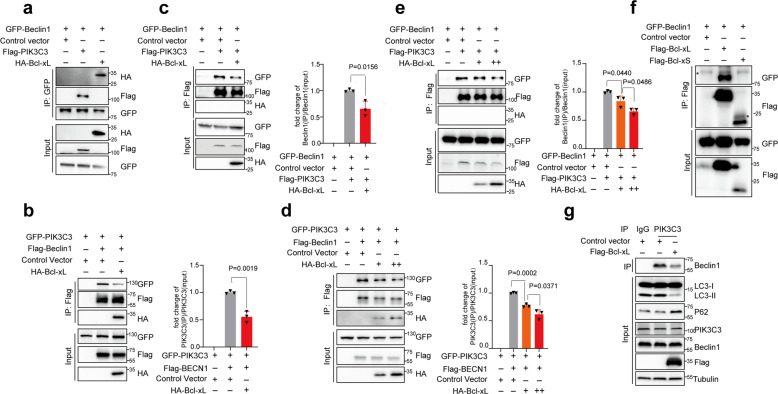
SRSF1-mediated splicing of Bcl-xL inhibits autophagy through disruption of the formation of the autophagy initiation complex. 293 T cells were co-transfected with different combinations of Beclin1, PIK3C3, and Bcl-xL expression vectors in **a**–**f**. **a** 293 T cells were co-transfected with pEGFP-C1-Beclin1 and control vector; or pEGFP-C1-Beclin1 and Flag-PIK3C3 expression vector; or pEGFP-C1-Beclin1 and HA-Bcl-xL expression vector, respectively. Co-immunoprecipitation assay was carried out with anti-GFP antibody and the precipitated complexes were measured by western blot with anti-HA, anti-Flag, or anti-GFP antibodies. **b** 293 T cells were co-transfected with pEGFP-C1-PIK3C3 and control vector; or pEGFP-C1-PIK3C3 and Flag-Beclin1 expression vector; or pEGFP-C1-PIK3C3, and Flag-Beclin1 and HA-Bcl-xL expression vectors. **c** 293 T cells were co-transfected with pEGFP-C1-Beclin1 and control vector; or pEGFP-C1-Beclin1 and Flag-PIK3C3 expression vector; or pEGFP-C1-Beclin1, and Flag-PIK3C3 and HA-Bcl-xL expression vectors. **d** 293 T cells were co-transfected with pEGFP-C1-PIK3C3 and control vector; or pEGFP-C1-PIK3C3, and Flag-Beclin1 expression vector; or pEGFP-C1-PIK3C3, and Flag-Beclin1 vector, with increased amounts of HA-Bcl-xL expression vectors (100 ng and 500 ng) respectively. **e** 293 T cells were co-transfected with pEGFP-C1-Beclin1 and control vector; or pEGFP-C1-Beclin1, and Flag-PIK3C3 expression vector; or pEGFP-C1-Beclin1, and Flag-PIK3C3 expression vector, with increased amounts of HA-Bcl-xL expression vectors (100 ng and 500 ng) respectively. Three experiments were conducted respectively and the mean ± SD of the relative ratio of the precipitated Beclin1 or PIK3C3 were analyzed and plotted in **b**–**e**. **f** 293 T cells were co-transfected with pEGFP-C1-Beclin1 and control vector; or pEGFP-C1-Beclin1 and Flag-Bcl-xL expression vector; or pEGFP-C1-Beclin1 and Flag-Bcl-xS expression vector. Co-immunoprecipitation assay was carried out with anti-Flag M2 beads and the precipitated complexes were measured by Western blot with anti-HA, anti-Flag, or anti-GFP antibodies in **b**–**f**. **g** H1299 cells with overexpression of Bcl-xL were used for the immunoprecipitation experiments. Immunoprecipitation assay was performed with anti-PIK3C3 antibody and the endogenously precipitated complexes were measured by western blot assay with anti-Beclin1 antibody. The lysate was applied to examine the autophagy status

Beclin1 interacts with PIK3C3 to form a complex that plays key roles in autophagy initiation.^41^ Bcl-xL has been shown to interact with Beclin1 and inhibit Beclin1-mediated autophagy;^42^ however, the detailed mechanisms are still elusive. To determine whether SRSF1-mediated splicing of Bcl-xL inhibits autophagy through interfering with the interaction between Beclin1 and PIK3C3, we performed co-immunoprecipitation experiments between Beclin1 and PIK3C3, as well as Bcl-xL, and confirmed that Beclin1 could interact with both PIK3C3 and Bcl-xL in both 293 T cells and H1299 cells (Fig. 4a, Supplementary Fig. 4a, b). Importantly, the interaction between Bcl-xL and Beclin1 disrupted the interaction of Beclin1 and PIK3C3, as less PIK3C3 protein was precipitated by anti-Flag antibody upon overexpression of Bcl-xL (Fig. 4b, Supplementary Fig. 4b). Ectopic expression of Bcl-xL also reduced the amount of Beclin1 in the precipitation by Flag-PIK3C3 as compared to control (Fig. 4c, Supplementary Fig. 4c). Further co-IP assay with increased amounts of Bcl-xL indicated that the interaction between Beclin1 and PIK3C3 was dose dependently disrupted by Bcl-xL (Fig. 4d, e, Supplementary Fig. 4d, e). Importantly, the short isoform, Bcl-xS, lost the interaction with Beclin1 (Fig. 4f, Supplementary Fig. 4f). To further corroborate our data, we conducted immunoprecipitation assay with anti-PIK3C3 antibody to examine whether the endogenous interaction between Beclin1 and PIK3C3 could be disrupted by Bcl-xL, we found that the expression of Bcl-xL indeed reduced the amount of Beclin1 precipitated by endogenous PIK3C3, thereby inhibiting autophagy (Fig. 4g). Strikingly, we found that Bcl-xL binds to the N-terminal BD domain of Beclin1, whereas PIK3C3 binds to the CCD-ECD domain of Beclin1 (Supplementary Fig. 4g). Although Bcl-xL is binding to a different region of Beclin1 as PIK3C3, it might affect the structure of Beclin1, thereby disrupting the interaction between Beclin1 and PIK3C3. Collectively, our data indicate that Bcl-xL, but not Bcl-xS, is able to impair the formation of the Beclin1-PIK3C3 complex, suggest that SRSF1-mediated splicing switch from Bcl-xS to Bcl-xL is crucial for inhibition of autophagy initiation. Most importantly, these results were validated in both 293 T and H1299 cells.

### SRSF1 interacts with PIK3C3 to disrupt the Beclin1-PIK3C3 complex and suppresses autophagy

**Fig. 5:**
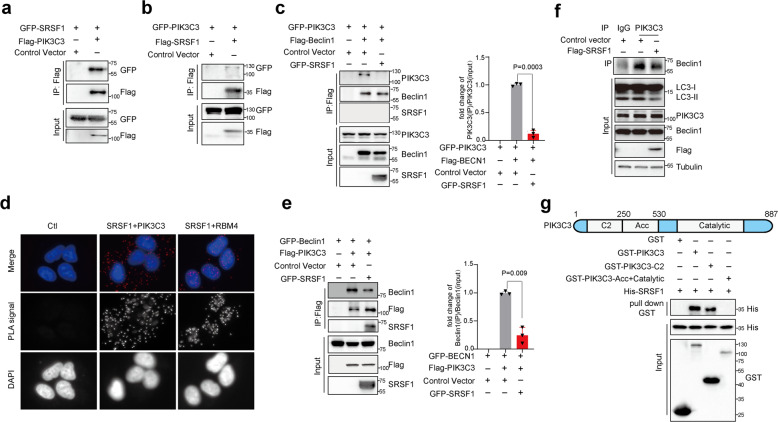
SRSF1 interacts with PIK3C3 to disrupt the Beclin-PIK3C3 complex and suppresses autophagy. 293 T cells were co-transfected with different combinations of Beclin1, PIK3C3, and SRSF1 expression vectors in **a**–**c** and **e**. **a** 293 T cells were co-transfected with pEGFP-C1-SRSF1 and control vector; or pEGFP-C1-SRSF1 and Flag-PIK3C3 expression vector. **b** 293 T cells were co-transfected with pEGFP-C1-PIK3C3 and control vector; or pEGFP-C1-PIK3C3 and Flag-SRSF1 expression vector. **c** 293 T cells were co-transfected with pEGFP-C1-PIK3C3 and control vector; or pEGFP-C1-PIK3C3 and Flag-Beclin1 expression vector; or pEGFP-C1-PIK3C3, Flag-Beclin1, and GFP-SRSF1 expression vectors. **d** Proximity ligation assay (PLA) was performed to examine the endogenous interaction between SRSF1 and PIK3C3 in A549 cells. The examination of the endogenous interaction between SRSF1 and RBM4 was assayed as a positive control. PLA signals were shown in red and the nuclei were demonstrated in blue. **e** 293 T cells were co-transfected with pEGFP-C1-Beclin1 and control vector; or pEGFP-C1-Beclin1, and Flag-PIK3C3 expression vector; or pEGFP-C1-Beclin1, Flag-PIK3C3 and GFP-SRSF1 expression vectors. Immunoprecipitation assay was performed with anti-Flag M2 beads and the precipitated complexes were analyzed by western blot with anti-Beclin1, anti-PIK3C3, or anti-SRSF1 antibodies in **c**, **e**. Three experiments were conducted and the mean ± SD of the relative ratio of the precipitated Beclin1 or PIK3C3 were analyzed and plotted in **c**, **e**. **f** H1299 cells with overexpression of SRSF1 were used for the immunoprecipitation experiments. Immunoprecipitation assay was performed with anti-PIK3C3 antibody and the precipitated complexes were measured by western blot assay with anti-Beclin1 antibody. The lysate was applied to examine the autophagy status. **g** GST pull-down assays to analyze direct binding of recombinant human GST-tagged PIK3C3, GST-tagged PIK3C3 C2 domain, GST-tagged PIK3C3 Acc Catalytic domain and His-tagged SRSF1. The *p* values were calculated by t-test in panels **c**, **e**

Unexpectedly, in both 293 T cells and H1299 cells, we found that SRSF1 was co-immunoprecipitated with PIK3C3 (Fig. 5a, b, Supplementary Fig. 5a, b) but not with Beclin1 (Fig. 5c, Supplementary Fig. 5c), suggesting that SRSF1 could only interact with PIK3C3. Subsequently, we performed Proximity Ligation Assay (PLA), which is a powerful tool that allows in situ detection of endogenous protein interactions with high specificity and sensitivity. Consistent with the co-immunoprecipitation results, SRSF1 can indeed interact with endogenous PIK3C3 mostly in cytoplasm (Fig. 5d). As a positive control, SRSF1 was found to interact with RBM4 in nucleus as reported previously (Fig. 5d).^38^ Most importantly, SRSF1 appears to compete with Beclin1 to bind PIK3C3 as ectopic expression of SRSF1 decreased the amount of PIK3C3 protein that was precipitated by Beclin1 (Fig. 5c, Supplementary Fig. 5c). Similarly, the levels of Beclin1 precipitated by PIK3C3 were reduced by the expression of SRSF1 as well (Fig. 5e, Supplementary Fig. 5d). We subsequently performed immunoprecipitation assay with anti-PIK3C3 antibody to examine whether the endogenous interaction between Beclin1 and PIK3C3 could be dissociated by SRSF1. Our data demonstrated that SRSF1 disrupts the endogenous interaction of Beclin1 and PIK3C3, thus to inhibit autophagy (Fig. 5f). Most importantly, we further proved that SRSF1 is able to directly interact with PIK3C3 in an in vitro binding assay (Fig. 5g). Mechanistically, we found that SRSF1 is capable of binding to the C2 region of PIK3C3 that is the same binding region as Beclin1 (Fig. 5g).^43^ Therefore, in addition to inducing splicing switch from Bcl-xS to Bcl-xL to suppress autophagy, SRSF1 could also directly disrupt the formation of Beclin1-PIK3C3 complex by binding to PIK3C3 in both 293 T and H1299 cells, thereby inhibiting initiation of autophagy.

### SRSF1 is degraded by starvation-induced autophagy through LC3 conjugation

**Fig. 6:**
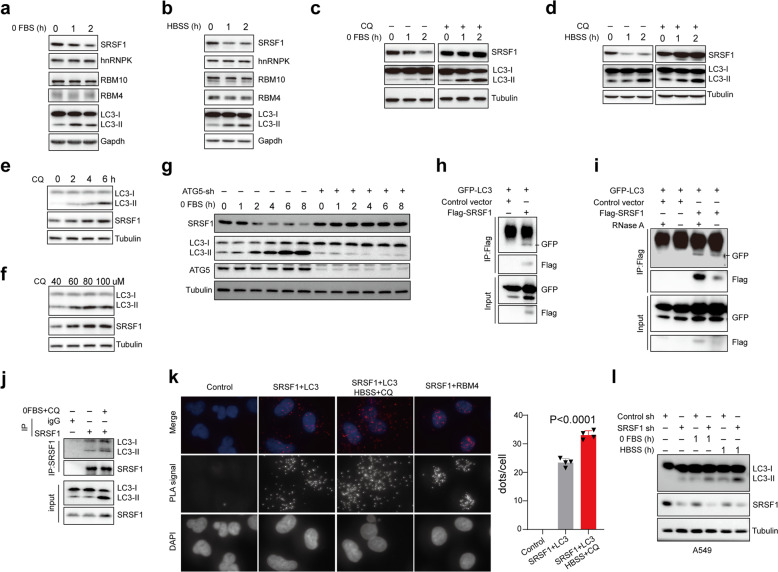
SRSF1 is degraded by starvation-induced autophagy through LC3 conjugation. **a**, **b** A549 cells were treated with serum-free medium or HBSS medium for the indicated time. Proteins were isolated from the resulting cells and the levels of SRSF1, hnRNP K, RBM10, RBM4, and LC3 were determined with a western blot assay. **c** A549 cells were treated with serum-free medium for the indicated time without or with CQ treatment (100 µM). The cell lysates were collected to examine the protein levels of SRSF1 and LC3 using a western blot assay. **d** A549 cells were treated with HBSS medium for the indicated time without or with CQ treatment (100 µM). The cell lysates were isolated to measure the protein levels of SRSF1 and LC3 by the western blot approach. **e** A549 cells were treated with 40 µM CQ for 0, 2, 4, and 6 h, respectively. The protein levels of LC3 and SRSF1 were measured with a western blot assay. **f** A549 cells were treated with different concentrations of CQ, respectively, (40, 60, 80, and 100 µM) for 4 h. The protein levels of LC3 and SRSF1 were measured with a western blot assay. **g** HeLa cells with stable knockdown of ATG5 or control were treated with serum-free medium for the indicated time. The cell lysates were isolated to measure the protein levels of SRSF1, LC3-II, and ATG5 by western blot. **h** 293 T cells were co-transfected with pEGFP-C1-LC3 and control vector; or pEGFP-C1-LC3 and Flag-SRSF1 expression vector. Co-immunoprecipitation assay was carried out with anti-Flag M2 beads and the precipitated complexes were analyzed by a western blot assay with anti-GFP, or anti-Flag antibodies. **i** 293 T cells were co-transfected with pEGFP-C1-LC3 and control vector; or pEGFP-C1-LC3 and Flag-SRSF1 expression vector. Co-immunoprecipitation assay was performed with anti-Flag M2 beads under the condition with or without RNase A treatment and the precipitated complexes were analyzed by a western blot experiment with anti-GFP, or anti-Flag antibodies. **j** Immunoprecipitation was carried out with SRSF1 antibody. Endogenous LC3 was measured using western blots in immunoprecipitated complexes. **k** Proximity ligation assay (PLA) was performed to examine the endogenous interaction between SRSF1 and LC3 in A549 cells in the absence or presence of HBSS and CQ for 4 h. The examination of the endogenous interaction between SRSF1 and RBM4 was assayed as a positive control. PLA signals were shown in red and the nuclei were demonstrated in blue. Three experiments were carried out and the number of dots of PLA signals were counted by using Image J and represented with mean ± SD. **l** A549 cells with stable knockdown of SRSF1 or control were treated with or without serum-free, or HBSS medium for 1 h. Cell lysates isolated from the treated cells were applied to western blots to determine the protein levels of LC3 and SRSF1

Interestingly, we observed an evident reduction of SRSF1 protein levels in response to starvation (serum-free medium, or HBSS treatment) (Fig. 6a, b) or Rapamycin treatment (Supplementary Fig. 6a), and such decrease of SRSF1 is associated with an accumulation of LC3-II, while other splicing factors such as hnRNP K, RBM10, and RBM4, did not change in expression (Fig. 6a, b). Consistent with this observation, the level of Bcl-xL was decreased accordingly with the serum-free medium of HBSS treatment (Supplementary Fig. 6b). Moreover, the starvation-induced decrease in SRSF1 protein was largely prevented by CQ treatment (100 µM) (Fig. 6c, d), but not the proteosome inhibitor MG132 (Supplementary Fig. 6c). Additionally, the level of SRSF1 was elevated accordingly in a time course manner with CQ treatment (Fig. 6e). The accumulation of SRSF1 was also observed with increased concentrations of CQ treatment (Fig. 6f), indicating that SRSF1 might be degraded by starvation-induced autophagy. In addition to nutrient starvation, autophagy can also be induced with other oxidative stresses,^44,45^ we therefore treated cells with hydrogen peroxide (H_2_O_2_) or sodium arsenite, and verified that the level of SRSF1 was dramatically decreased in such oxidative stresses-induced autophagy as well (Supplementary Fig. 6d). To further prove that SRSF1 is degraded through autophagy, we depleted ATG5, a factor required for autophagic vesicle formation, and also found knockdown of ATG5 could inhibit the starvation-induced reduction of SRSF1, suggesting that induction of autophagy itself leads to decreased SRSF1 protein (Fig. 6g, Supplementary Fig. 6e).

In support of the notion that starvation-induced SRSF1 degradation is mediated by LC3 conjugation, SRSF1 was identified to interact with LC3 in a co-IP assay (Fig. 6h). The interaction between SRSF1 and LC3 was independent of RNA, as the interaction is still existing with RNase A treatment (Fig. 6i). Starvation also promoted the interaction between endogenous SRSF1 and LC3 (Fig. 6j). In addition, we performed Proximity Ligation Assay (PLA) to further examine the endogenous protein interaction between SRSF1 and LC3. As expected, our data further revealed that SRSF1 can indeed interact with endogenous LC3 mostly in cytoplasm, and the treatment of HBSS and CQ could enhance their interactions (Fig. 6k). As a positive control, SRSF1 was found to interact with RBM4 in nucleus as reported previously (Fig. 6k).^38^ The colocalization of SRSF1 with LC3 was also validated with confocal microscopy assay (Supplementary Fig. 6g). Finally, starvation-induced autophagy was enhanced in the absence of SRSF1 (Fig. 6l). Altogether, our data suggest that SRSF1 is degraded through autophagy in response to starvation, and reduction of SRSF1 further enhances autophagy, forming a positive feedback loop to promote autophagy.

### Reduced SRSF1 inhibits Gefitinib-resistant cancer cell progression partially through activating autophagy

**Fig. 7:**
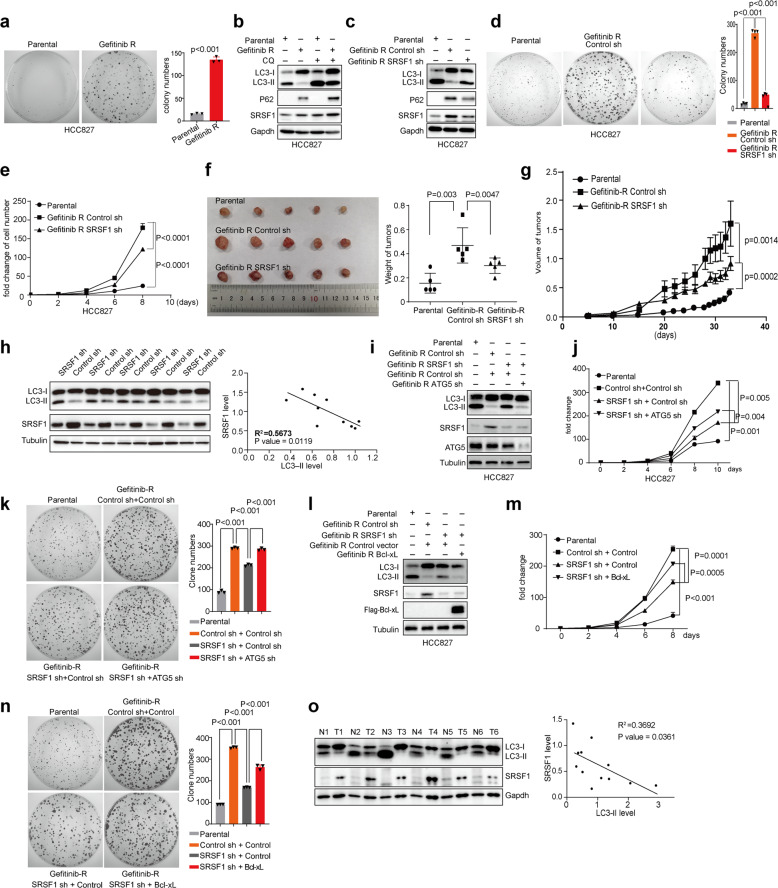
Reduced SRSF1 inhibits Gefitinib-resistant cancer cell progression by activating autophagy. **a** The growth ability of parental and Gefitinib-resistant HCC827 cells were examined by colony formation assay. Representative pictures of the whole plates from triplicate experiments are shown. The mean ± SD of colony numbers were plotted, with *p* values calculated by *t*-test. **b** The parental and Gefitinib-resistant HCC827 cells were treated without or with CQ (40 µM for 2 h). The protein levels of SRSF1, LC3, and p62 in the parental and Gefitinib-resistant HCC827 cells were measured with western blots. **c** The parental, and Gefitinib-resistant HCC827 cells with stable knockdown of SRSF1 or control were applied to examine the protein levels of LC3, p62, and SRSF1 with western blots. (**d**) The growth abilities of the parental, Gefitinib-resistant HCC827 cells stably depleted SRSF1 or control were determined by colony formation assay. Representative pictures of the whole plates from triplicate experiments are shown. The mean ± SD of colony numbers were plotted, with *p* values calculated by *t*-test. **e** The parental, Gefitinib-resistant HCC827 cells stably depleted SRSF1 or control were grown for 8 days, with cell numbers counted every 2 days. The changes of cell numbers were compared to day 0. The mean ± SD from three experiments was plotted. **f** The parental HCC827 cells, and Gefitinib-resistant HCC827 cells stably knocked down SRSF1 or control were subcutaneously injected into the flank of nude mice. Each group contained five mice. Pictures of the tumors removed after 32 days were shown. Tumors were weighed and plotted. **g** The average sizes of xenograft tumors were measured every four or 2 days (*n* = 5, error bars indicate ±SD, *p* < 0.05 by *t*-test). **h** Proteins isolated from tumors removed from mice were assayed by western blot approach to examine the levels of LC3 and SRSF1. The correlation of the levels of SRSF1 and LC3-II were plotted. **i** The parental, Gefitinib-resistant HCC827 cells with stable knockdown of control, SRSF1, SRSF1, and ATG5, were collected to determine the protein levels of LC3, ATG5, and SRSF1 using western blot assay. **j** The parental, Gefitinib-resistant HCC827 cells stably knocked down SRSF1, SRSF1, and ATG5, or control were grown for 10 days, with cell numbers counted every two days. The changes of cell numbers were compared to day 0. The mean ± SD from three experiments was plotted. **k** The growth abilities of the parental, Gefitinib-resistant HCC827 cells with stable depletion of SRSF1, SRSF1, and ATG5, or control were determined by colony formation assay. Representative pictures of the whole plates from triplicate experiments are shown. The mean ± SD of colony numbers were plotted, with *p* values calculated by *t*-test. **l** The parental, Gefitinib-resistant HCC827 cells with stable knockdown of control or SRSF1, overexpression of Bcl-xL, re-expression of Bcl-xL upon depletion of SRSF1, were collected to determine the protein levels of LC3, Flag-Bcl-xL and SRSF1 using western blot assay. **m** The parental, Gefitinib-resistant HCC827 cells with stable knockdown of control or SRSF1, overexpression of Bcl-xL, re-expression of Bcl-xL upon depletion of SRSF1, were grown for 8 days, with cell numbers counted every two days. The changes of cell numbers were compared to day 0. The mean ± SD from three experiments was plotted. **n** The growth abilities of the parental, Gefitinib-resistant HCC827 cells with stable knockdown of control or SRSF1, overexpression of Bcl-xL, re-expression of Bcl-xL upon depletion of SRSF1, were determined by colony formation assay. Representative pictures of the whole plates from triplicate experiments are shown. The mean ± SD of colony numbers were plotted, with *p* values calculated by *t*-test. **o** LC3 and SRSF1 levels from six paired NSCLC tumors (T) and normal (N) tissues were evaluated by western blots. The correlation between the levels of SRSF1 and LC3 was plotted

Autophagy has been previously shown to sensitize lung cancer cells to chemotherapeutics.^46,47^ Meanwhile, SRSF1 promotes lung cancer cell progression.^48^ Therefore, we next investigated whether SRSF1-mediated autophagy inhibition is involved in regulating the proliferation of drug-resistant cancer cells. Consistent with this possibility, Gefitinib-resistant HCC827 lung cancer cells, which had a much faster growth rate as compared to the parental HCC827 cells (Fig. 7a), also displayed elevated expression of SRSF1 (Fig. 7b). However, no matter whether cells were treated with or without CQ (40 µM for 2 h), the accumulation of LC3-II was noticeably reduced in Gefitinib-resistant cells as compared to the parental cells (Fig. 7b). Crucially, stable knockdown of SRSF1 increased the accumulation of LC3-II in Gefitinib-resistant cells (Fig. 7c), sensitized the resistant cancer cells to Gefitinib as compared to control (Supplementary Fig. 7a), and inhibited the growth of these Gefitinib-resistant cancer cells as judged by colony formation and growth curve assays (Fig. 7d, e), suggesting depletion of SRSF1 could suppress Gefitinib-resistant cancer cell proliferation at least partially through activating autophagy. In addition, the xenografts with parental HCC827 lung cancer cells developed the smallest tumors, whereas xenografts with SRSF1-knockdown Gefitinib-resistant cells developed smaller tumors as compared to controls (Fig. 7f), and grew much slower than controls as well (Fig. 7g). Most importantly, the level of LC3-II in most of the xenograft tumors with decreased SRSF1 cells was higher than tumors with control cells that have increased SRSF1 expression, indicating the level of LC3-II is negatively correlated to the level of SRSF1 (R^2^ = 0.5673, *p* = 0.0119) (Fig. 7h).

To further confirm that SRSF1-knockdown could inhibit Gefitinib-resistant cancer cell progression through activation of autophagy, we knocked down ATG5, a factor required for autophagic vesicle formation,^49^ in SRSF1-reduced Gefitinib-resistant HCC827 cells (Fig. 7i). As expected, knockdown of ATG5 partially reversed the growth inhibition resulted from SRSF1 reduction as judged by growth curve and colony formation assays (Fig. 7j, k). To further corroborate our conclusion, we knocked down ATG7, another key player in autophagy regulation, and found that decreased ATG7 in SRSF1-downregulated Gefitinib-resistant cancer cells could also partially reverse depleted SRSF1-induced suppression of cancer cell proliferation, thereby promoting cancer cells growth (Supplementary Fig. 7b–e). Similar results were also obtained in Paclitaxel-resistant HeLa cells as judged by colony formation assay (Supplementary Fig. 7f). Therefore, these results indicate that the reduced level of SRSF1 significantly inhibits Gefitinib-resistant cancer cell progression at least partially through activating autophagy.

Since SRSF1 reduction promotes autophagy initiation and inhibits Gefitinib-resistant cancer cell progression and tumorigenesis partially through suppressing splicing of Bcl-xL, we tested if co-expression of Bcl-xL could overturn SRSF1-knockdown-induced tumor suppression in Gefitinib-resistant cancer cells. We stably transfected control and SRSF1 depleted HCC837 Gefitinib-resistant cells with constructs expressing Bcl-xL or control vector (Fig. 7l). Strikingly, re-expression of Bcl-xL in SRSF1 knocked down cells partially rescued SRSF1 decrease-induced accumulation of LC3-II, and cell growth defect (Fig. 7l–n). Such phenotypical rescue indicates that SRSF1-knockdown-induced splicing switch from Bcl-xL to Bcl-xS promotes the autophagy initiation. In addition to regulating autophagy, Bcl-xL is an antiapoptosis factor. We also observed that depleted SRSF1 reduced the level of Bcl-xL, thereby inducing apoptosis as judged by an increase of cleaved-PARP and decrease of pro-PARP (Supplementary Fig. 7g). Moreover, SRSF1 also regulates the splicing of some other cancer-related genes, such as BIM, BIN, and MCL1 (Supplementary Fig. 7h), which might account for that ectopic expression of Bcl-xL only partially rescues the phenotype. Altogether, our data suggest that knockdown of SRSF1 could inhibit Gefitinib-resistant cancer cells progression at least in part via activating autophagy.

To assess the clinical relevance of SRSF1-regulated autophagy in tumorigenesis, we measured the levels of SRSF1 and LC3-II in surgically collected paired NSCLC samples and adjacent normal lung tissues from six patients. We found that all of the primary NSCLC specimens have a noticeable increase of SRSF1 protein but decrease of LC3-II expression as compared to paired normal lung tissues, indicating that the level of LC3-II is negatively correlated to the level of SRSF1 in patient samples (R^2^ = 0.3692, *p* = 0.0361) (Fig. 7o).

## Discussion

Autophagy is a crucial lysosome degradation pathway that is involved in development and cancer progression,^50^ which plays a tumor-suppressive or tumor-promoting role in distinct contexts and stages of cancer progression. Autophagy has been widely established as a tumor-suppressive mechanism, as defective autophagy has been closely associated with genomic instability, tumorigenesis, and malignant transformation.^51,52^ For example, mice having monoallelic deletion of the autophagy-related gene beclin1 develop spontaneous tumors. Allelic loss of beclin1 was also observed in 40–75% of breast, ovarian, and prostate cancers.^53–55^ Therefore, evidence suggests that Beclin1 induces autophagy to function as a tumor suppressor. In addition, Phosphatase and tensin homolog (PTEN) has been shown to promote autophagy in HT-29 colon cancer cells,^56^ and CK1α could suppress lung tumor growth by stabilizing PTEN and inducing autophagy.^57^ Microtubule-associated protein 1 small form (MAP1S) enhances autophagy to inhibit tumorigenesis.^58^ Importantly, MAP1S-interacting Ras association domain family 1 isoform A (RASSF1A) could also activate autophagy initiation and maturation to suppress hepatocellular carcinoma progression.^59^ Altogether, autophagy has opposite and context-dependent functions in tumor progression, and our results support that activation of autophagy is a therapeutic strategy to suppress tumorigenesis.

**Fig. 8:**
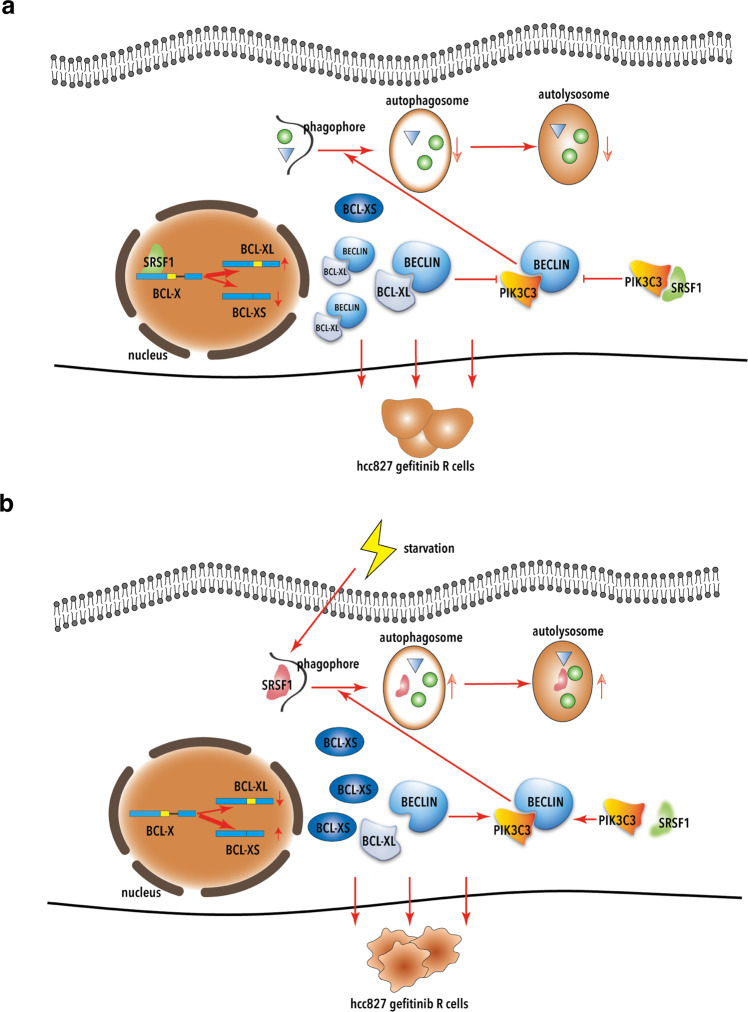
Regulation of SRSF1 in autophagy in cancer cells. **a** In normal conditions, SRSF1 disrupts the interaction between Beclin1 and PIK3C3 through promoting the production of Bcl-xL and directly binding to PIK3C3, thereby suppressing autophagy and stimulating gefitinib-resistant cancer cell proliferation. **b** Under starvation conditions, SRSF1 is degraded via starvation-induced autophagy, thus to stimulate the formation of Beclin1-PIK3C3 complex and activate autophagy. The resulting gefitinib-resistant cancer cells will be eliminated through autophagy

Many factors have been demonstrated to play vital roles in autophagy process, however, whether alternative splicing participates in autophagy regulation is still largely elusive. Here we report a model in which the alternative splicing plays a key regulatory role in mediating activation of autophagy. We showed that, under normal condition, the splicing factor SRSF1 binds to a potential binding site, which is similar to the reported SRSF1 motifs,^39^ in the pre-mRNA of Bcl-x, to promote the splicing switch of Bcl-x from the short isoform towards the long isoform, thereby dissociating the Beclin1-PIK3C3 complex to inhibit autophagy initiation. In addition, SRSF1 itself directly interacts with PIK3C3 to disrupt the interaction between Beclin1 and PIK3C3, thus to suppress autophagy initiation as well. Therefore, the Gefitinib-resistant cancer cells will proliferate (Fig. 8a). However, under the starvation condition, SRSF1 is degraded through starvation-induced autophagy. Importantly, loss of SRSF1 promotes the production of the short isoform of Bcl-x, which does not bind to Beclin1, leading to the release of Beclin1 and activation of autophagy. Moreover, reduction of SRSF1 will stimulate the formation of Beclin1-PIK3C3 complex, thereby activating autophagy. The resulting Gefitinib-resistant cancer cells will be eliminated through autophagy (Fig. 8b). Taken together, these data provide a novel mechanism on how alternative splicing affects cancer progression through regulating autophagy process.

Interestingly, the level of SRSF1 is decreased in the starvation-induced autophagy (Fig. 6a), In addition, the reduced level of SRSF1 is further validated in hydrogen peroxide-, and sodium arsenite-induced autophagy (Supplementary Fig. 6a). Altogether, these data support that SRSF1 could be degraded by nutrient starvation-induced autophagy, as well as oxidative stress-induced autophagy.

SRSF1 has been previously identified as a potential oncogene that promotes tumorigenesis in multiple cancers.^28,48,60^ Additionally, SRSF1 is a key member in SR-protein family, which is involved in a variety of biological functions, including alternative splicing regulation, translation control, RNA transportation, and nonsense-mediated RNA decay.^61–64^ In this study, we demonstrated autophagosomes were significantly increased in SRSF1-reduced cells. Whereas overexpression of SRSF1 led to a substantially decreased accumulation of LC3-II and autophagosomes amount in several cancer cell lines. Importantly, we discovered that SRSF1 could interact with PIK3C3 to disrupt the association of Beclin1 and PIK3C3 complex, thereby inhibiting autophagy initiation. Conversely, decrease of SRSF1 switches the splicing of Bcl-x from the long isoform to the short isoform, thus to suppress the Bcl-xL-induced prevention of the autophagy initiation. Together, knockdown of SRSF1 inhibits cancer progression partially through activating autophagy, providing a new mechanism for SRSF1 in tumorigenesis.

Previously, Bcl-xL has been shown to interact with Beclin1 to suppress autophagy;^35^ however, the detailed mechanisms are still unknown. In this study, we demonstrated that Bcl-xL could interact with Beclin1 to dissociate the Beclin1 and PIK3C3 complex, which is an important autophagy initiation complex. Importantly, the short isoform Bcl-xS lost the interaction with Beclin1, thereby failing to separate the Beclin1 and PIK3C3 complex. Therefore, we can conclude that Bcl-xL directly interacts with Beclin1 to disrupt the Beclin1 and PIK3C3 complex, which in turn inhibits the autophagy process.

More than 90% of human genes undergo alternative splicing to generate protein diversity.^18^ It is possible that alternative splicing, particularly splicing factors, might play critical roles in autophagy regulation. In our screening data, we also found that, in addition to SRSF1, several other key splicing factors might also regulate autophagy, including SRSF9, hnRNPF, and so on. In our future study, we will also investigate whether other splicing factors are involved in regulating autophagy, as well as the detailed molecular mechanisms. Since aberrant alternative splicing leads to cancer, this study might provide more evidence to determine the role of deregulated splicing and autophagy in tumorigenesis.

Autophagy is a multistep and highly dynamic process. Several key regulators in autophagy also undergo alternative splicing to produce different isoforms with distinct functions, such as Beclin1 for example.^30^ Beclin1 has a short isoform, Beclin1s, which is produced by alternative splicing. Importantly, unlike unspliced BECN1 that is essential for nonselective macroautophagy induction, BECN1s is indispensable for mitochondria-selective autophagy. Therefore, investigation of aberrant splicing and the regulatory mechanisms in autophagy might provide new insights into the clinical application of autophagy-related cancer therapeutics.

## Materials and methods

### Cell culture and treatment

Human lung cancer A549, NCI-H1299, and H460 cell lines were obtained from the American Type Culture Collection (Manassas, VA, USA) and were maintained in F-12K and RPMI-1640 medium supplemented with 10% Fetal Bovine Serum (FBS) respectively. To stably overexpress SRSF1 or knockdown SRSF1 in A549 and H1299 cells, lentiviral vectors were used. We transfected 293 T cells with pCDH-Flag-SRSF1(pCDH-Flag empty vector as control) or pLKO.1-SRSF1 (pLKO.1 empty vector as control) together with PAX2 and PMD2 according to the manufacture’s protocols. The supernatant media containing virus were collected by centrifugation to remove any cellular contaminant. Further, A549 and H1299 cells were infected with the viral particles, and the stably integrated cells were selected with 5 µg/ml puromycin for 5 days. Then cells were maintained in medium containing 2 µg/ml puromycin at 37°C in a humidified incubator with 5% CO_2_. All the stable cell lines were confirmed by western bolts before further analysis.

For induction of autophagy, cells were starved by incubating in HBSS medium (GIBCO) or medium without FBS, or treated with Rapamycin. Lysosomal degradation was inhibited by 40 µM chloroquine (CQ, Sigma–Aldrich) for 2 or 4 h.

### Fluorescence microscopy

For monitoring autophagy, A549 (or H1299) cells with SRSF1 overexpression or knockdown were transfected with GFP-tagged LC3 for 24 h using Lipofectamine 3000 (Thermo Fisher). Images were obtained under a confocal microscope (Leica TCS SP5 ×) with a ×60 oil objective. The number of GFP-LC3 puncta per cells were analyzed.

For examining autophagic flux, A549 (or H1299) cells with SRSF1 overexpression or knockdown were transfected with mRFP-GFP-tagged LC3 for 24 h using Lipofectamine 3000 (Thermo Fisher). Then Images were acquired using a confocal microscope (Leica TCS SP5 ×) with a ×60 oil objective. Autophagosomes were detected as RFP^+^GFP^+^ (yellow dot), while mature autolysosomal organelles were detected as RFP^+^GFP^−^(red-only dot).

For immunofluorescence staining, cells were cultured on sterile glass cover slips and washed briefly with PBS and then fixed with 4% paraformaldehyde for 10 min. Cells were permeabilized in 0.2% Triton X-100 for 10 min, and blocked in 3% bovine serum albumin for 1 h. Then, the cells were stained with primary antibodies against LC3 (Sigma L7543) and SRSF1 (Santa sc-33653). Nuclei were stained with DAPI. Cells were visualized and images were captured using a Leica TCS SP5 confocal microscope (Leica, Germany).

### Proximity ligation assay (PLA)

For proximity ligation assay, cells were cultured on sterile glass cover slips, and subsequently treated in the presence or absence of HBSS and CQ for 4 h. Cells were washed briefly with PBS and then fixed with 4% paraformaldehyde for 10 min, permeabilized in 0.2% Triton X-100 for 10 min at 4 C°. Then the cells were subsequently processed as per manufacturer’s instructions (DUO92101 Sigma–Aldrich). Primary antibodies used as follows: LC3 (Sigma L7543), SRSF1 (Santa sc-33653), RBM4 (Proteintech 11614-1-AP). Primary antibodies were diluted to 1:100. Cells were visualized and images were captured using Leica microscope (Leica Mi8, Germany).

### RT-PCR

Total RNA was extracted from cells using Trizol reagent (Invitrogen) according to the manufacturer’s instructions. Genome DNAs were removed by 30 min DNase I (Takara) treatment at 37 °C and then heat inactivation using 50 nM EDTA. Total RNA (2 µg) was then reverse-transcribed with PrimeScript RT reagent kit (Takara) with random primer, and 1 ul of the cDNA was used as the template for PCR amplification. RT-PCR products were separated on 3% gels. The amount of each splicing isoform was measured by comparison of the integrated optical density of detected bands using the Image J.

### Western blot

Cells were harvested with RIPA lysis buffer containing 1 mM Na_3_VO_4_, 1 mM Cocktail and 1 mM PMSF. Cell debris was removed by centrifugation. The protein samples (30 µg) were boiled of 5 min in 1× SDS sample buffer and fractionated by 12% SDS-PAGE and transferred to PVDF membrane. The following antibodies were used: LC3 (Sigma L7543), SRSF1 (Santa sc-33653), BCL-X (Santa sc-634), GFP (Roche 11814460001), Flag (Sigma), Tubulin (T5168). Bound antibodies were visualized with enhanced chemiluminescence (Tanon) by MiniChmei Chemiluminescence imager (SAGECREATION, Beijing).

### Colony formation assay

SRSF1 stable knockdown HCC827 cells were seeded in 60-mm dishes (500 cells per dish) and incubated at 37 °C,5% CO_2_ in humidified incubator for 10 days or two weeks (updated with fresh medium every 3 days). Each treatment was carried out in triplicate. Colonies were fixed with 4% paraformaldehyde and stained with crystal violet solution.

### Immunoprecipitation

For immunoprecipitation, cells were transfected with GFP-tagged LC3 and flag-SRSF1 using Lipofectamine 3000. After 24 h, cells were lysed in lysis buffer (50 mM Tris/HCl, pH 7.5, 150 mM NaCl, 1 mM EDTA, 1% Triton X-100, 1 mM Cocktail and 1 mM PMSF). After pretreatment with protein G–agarose (Roche), cellular lysates were incubated with Anti-FLAG M2 beads (Sigma) at 4 °C overnight. Then TBS (50 mM Tris/HCl, pH 7.5, 150 mM NaCl) with 0.1% Triton X-100 was used to wash the beads for five times then the immunocomplexes were subjected to immunoblot analyses with corresponding antibodies.

### Transmission electron microscopy

For ultrastructural analysis of autophagosome, transmission electron microscopy (TEM) was carried out. A549 and H1299 cells were fixed with 2.5% glutaraldehyde in 0.1 M PBS (pH 7.4) at 4 °C for 2.5 h, washed three times with 0.1 M PBS and post-fixed in 1% OsO_4_ for 2 h at 4 °C. The samples were subsequently dehydrated through an ethanol gradient and embedded in Spurr’s resin. Ultrathin sections were then collected and stained with either uranyl acetate or lead citrate and examined by a JEOL 1200EX transmission electron microscope.

### Xenograft assays

Fifteen 4-week-old female BALB/c nude mice were purchased from Vital River Laboratories (VRL) for in vivo tumorigenicity study. Mice were injected subcutaneously with 1 × 10^6^ parental HCC827 cells, and HCC827-Gefitinib-resistant cells stably knocking down SRSF1, and control. Five mice were used for each group. Mice were raised in the following 32 days. The mice were then monitored for tumor volume and overall health. The size of the tumor was determined by caliper measurement of the subcutaneous tumor mass every four or 2 days. Tumor volume was calculated according to the formula 1/2r_1_^2^r_2_, (r_1_ < r_2_). At the end of 32 days, all mice were sacrificed, and tumors were removed for further analysis. For all data points, five independent measurements were performed and means were used for calculation.

### Clinical tissues samples collection

Fresh lung cancer tissues and adjacent normal tissues were collected from patients with pathologically and clinically confirmed lung carcinomas. All of tissue specimens were kept in liquid nitrogen and sectioned for protein or mRNA extraction.

### ESEfinder analysis

To predict the binding sites of SRSF1 in Bcl-x gene, we used the online bioinformatic tool ESEfinder 3.0 (http://krainer01.cshl.edu/cgi-bin/tools/ESE3/esefinder.cgi?process=home). Briefly, we input the sequences of Bcl-x containing exon 2 and the 5′ extension region of exon 2 in the dialogue box. The potential binding motifs of SRSF1 will be predicted with a score. A high score usually presents a high binding possibility of SRSF1.

### In vitro binding assay

Plasmid expressing the recombinant proteins of PIK3C3 and SRSF1 were transformed into SoluBL21 *Escherichia coli* and then were induced by IPTG at 37 °C for 12 h. The bacteria were collected and then lysed by sonication. His-tagged SRSF1 were purified by Ni-NTA Agarose (QIAGEN, 30210). GST-tagged control, GST-tagged PIK3C3, GST-tagged PIK3C3 C2 domain, GST-tagged PIK3C3 Acc Catalytic domain were purified using Glutathione High Capacity Magnetic Agarose Beads (sigma, G0924). Immobilized GST-tagged proteins on Glutathione High Capacity Magnetic Agarose Beads were incubated with His-tagged SRSF1 at 4 °C. After removing the supernatant, the beads were washed with the wash buffer for 5 times. The target proteins were eluted with SDS loading buffer and detected by western blotting.

### Statistical analysis

Quantitative analysis of immunoblotting and RT-PCR was performed using ImageJ software. Differences between experimental groups were evaluated by *t*-test using Graphpad Prism 7 software package to analyze the expression and splicing changes. Statistical significance was based on a P-value of 0.05.

### Study approval

The Institutional Animal Care and Use Committee of the Dalian Medical University approved use of animal models in this study.

All human tumor tissues were obtained with written informed consent from patients or their guardians prior to participation in the study. The Institutional Review Board of the Dalian Medical University approved use of the tumor specimens in this study.

## Supplementary information

Supplemental material

## Data Availability

The authors declare that all the data supporting the findings of this study are available within the article and its supplementary information files.
