# Supplementary material for: SRSF1 inhibits autophagy through regulating Bcl-x splicing and interacting with PIK3C3 in lung cancer
Source: Signal Transduct Target Ther. 2021 Mar 5;6:108. doi: 10.1038/s41392-021-00495-6 (PMC7933324; doi:10.1038/s41392-021-00495-6)
Supplement: Supplementary file 1 — Supplemental material [file 41392_2021_495_MOESM1_ESM.pdf]

Supplementary Materials for

SRSF1 Inhibits Autophagy Through Regulating Bcl-x Splicing and Interacting with PIK3C3 in Lung Cancer

Yuesheng Lv[#], Wenjing Zhang[#], Jinyao Zhao[#], Bing Sun[#], Yangfan Qi, Haoyu Ji, Chaoqun Chen, Jinrui Zhang, Junxiu Sheng, Taishu Wang, Daniel Dominguez, Han Liu, Quentin Liu, Songshu Meng, Xiaoling Li, Yang Wang[*]

Correspondence to: yangwang@dmu.edu.cn

This PDF file includes:

Figures. S1 to S7

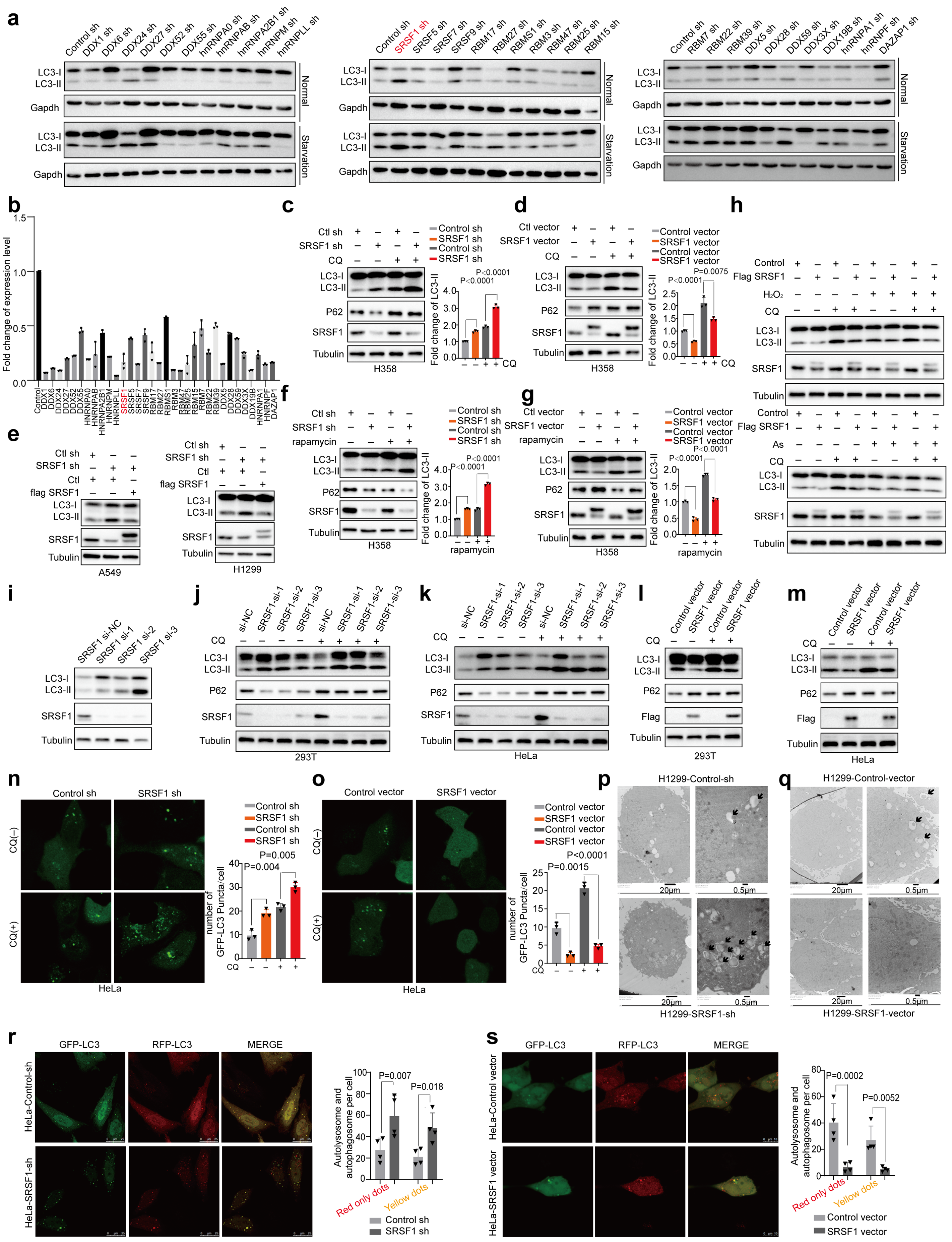
a
Control sh
DDX1 sh
DDX6 sh
DDX24 sh
DDX27 sh
DDX52 sh
DDX55 sh
hnRNPA0 sh
hnRNPAB sh
hnRNPA2B1 sh
hnRNPM sh
hnRNPLL sh
LC3-I
LC3-II
Gapdh
Normal
Starvation
SRSF1 sh
SRSF5 sh
SRSF7 sh
SRSF9 sh
RBM17 sh
RBM27 sh
RBMS1 sh
RBM3 sh
RBM47 sh
RBM25 sh
RBM15 sh
RBM7 sh
RBM22 sh
RBM39 sh
DDX5 sh
DDX28 sh
DDX59 sh
DDX3X sh
DDX19B sh
hnRNPA1 sh
hnRNPF sh
DAZAP1 sh
b
Fold change of expression level
c
Ctl sh
SRSF1 sh
CQ
P62
SRSF1
Tubulin
H358
Fold change of LC3-II
d
Ctl vector
SRSF1 vector
Control vector
h
Control
Flag SRSF1
H2O2
e
flag SRSF1
A549
H1299
f
rapamycin
g
As
i
SRSF1 si-NC
SRSF1 si-1
SRSF1 si-2
SRSF1 si-3
j
si-NC
SRSF1-si-1
SRSF1-si-2
SRSF1-si-3
293T
k
HeLa
l
Flag
m
n
CQ(−)
CQ(+)
number of GFP-LC3 Puncta/cell
o
p
H1299-Control-sh
H1299-SRSF1-sh
q
H1299-Control-vector
H1299-SRSF1-vector
r
GFP-LC3
RFP-LC3
MERGE
HeLa-Control-sh
HeLa-SRSF1-sh
Autolysosome and autophagosome per cell
Red only dots
Yellow dots
s
HeLa-Control vector
HeLa-SRSF1 vector

Figure. S1. SRSF1 inhibits autophagy in multiple conditions and cell lines. (a) The protein levels of LC3 were examined in A549 lung cancer cells with stable depletion of different splicing factors using distinct shRNAs under both starved and normal conditions. **(b)** The mRNA levels of tested splicing factors were examined by qRT-PCR in A549 cells stably down-regulated different splicing factors. **(c)** and **(d)** The protein levels of LC3, p62, and SRSF1 were examined in H358 cells with stable knockdown or overexpression of SRSF1 or control. Cells were treated without or with CQ (40 μM for 2 hours). The densities of signals were determined by densitometry and three experiments were carried out with mean +/- SD of relative fold change of LC3-II plotted. **(e)** The protein levels of LC3, and SRSF1 were examined in SRSF1 stably depleted A549 or H1299 cells with re-expression of SRSF1 or control. **(f)** and **(g)** H358 cells stably decreased or overexpressed SRSF1 or control were treated with Rapamycin, an inhibitor of mTOR pathway. The protein levels of LC3, p62, and SRSF1 were determined. The densities of signals were determined by densitometry and three experiments were carried out with mean +/- SD of relative fold change of LC3-II plotted. **(h)** Autophagy was induced by hydrogen peroxide (H_2O_2) or sodium arsenite in the absence or presence of CQ (40 μM for 2 hours) in A549 cells with stable expression of SRSF1 or control. The protein levels of LC3 and SRSF1 were determined. **(i)** The protein levels of LC3 and SRSF1 were measured in A549 cells with transient depletion of SRSF1 with a panel of siRNAs. **(j-k)** The protein levels of LC3, p62, and SRSF1 were measured in 293T and HeLa cells with transient knockdown of SRSF1 with siRNAs in the presence or absence of CQ treatment (40 μM for 2 hours). **(l-m)** The protein levels of LC3 and SRSF1 were examined in 293T and HeLa cells with overexpression of SRSF1 or control vector in the presence or absence of CQ treatment. **(n)** HeLa cells with stable decrease of SRSF1 or control were transfected with GFP-LC3. 24 hours after transfection, cells were treated with or

without CQ for 4 hours. The number of GFP-LC3 puncta per cell are represented with mean +/- SD. **(o)** HeLa cells stably overexpressed SRSF1 or control vector were transfected with GFP-LC3. 24 hours after transfection, cells were treated with or without CQ for 4 hours. The number of GFP-LC3 puncta per cell are represented with mean +/- SD. **(p)** and **(q)** The autophagosomes of H1299 cells with stable knockdown or overexpression of SRSF1 were examined with transmission electron microscopy (TEM). Representative pictures of 10000 and 20000-fold amplification were shown, and autophagosomes was indicated by black arrows. **(r)** and **(s)** HeLa cells with stable reduction or overexpression of SRSF1 were transfected with GFP-mRFP-LC3 to examine the expression of GFP and RFP by confocal microscope. The number of autophagosomes (yellow dots) and autolysosomes (red-only dots) per cell were quantified. The p values were calculated by t-test in all panels.

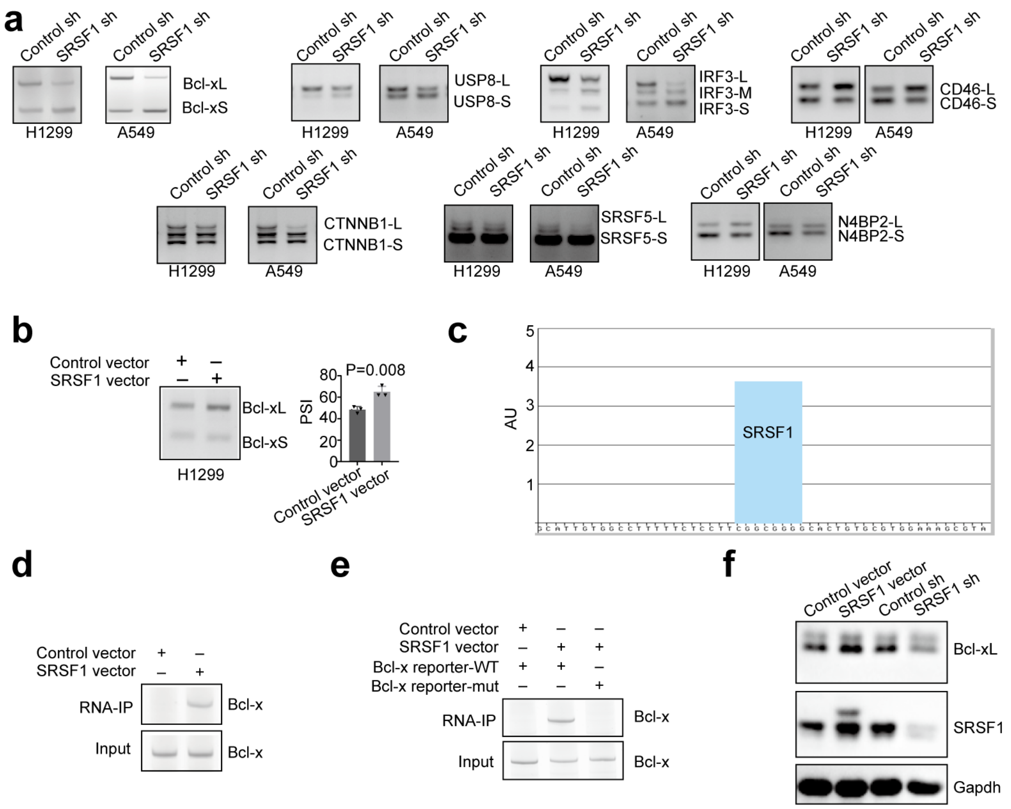

Figure. S2. SRSF1 regulates Bcl-x splicing. (a) The alternative splicing of Bcl-x, USP8, IRF3, CD46, CTNNB1, SRSF5, and N4BP2 was measured by RT-PCR when SRSF1 was knock down in H1299 and A549 cells. The representative gel figures were demonstrated. **(b)** The splicing of Bcl-x was examined in H1299 cells with stably overexpressed SRSF1 or control vector. **(c)** The potential SRSF1 binding site in Bcl-x pre-mRNA was predicted using the online ESFfinder tool. **(d)** Binding of Bcl-x pre-mRNAs with SRSF1 was examined by RNA-immunoprecipitation assay in 293T cells exogenously expressed FLAG-SRSF1 or control vector. **(e)** 293T cells were co-transfected with Flag-SRSF1 or control vector and the wild-type or mutant Bcl-x reporters, and subsequently immunoprecipitated with anti-Flag antibody. The co-precipitated RNAs were utilized to examine the level of Bcl-x by RT-PCR. A representative gel was demonstrated. **(f)** The protein levels of Bcl-xL and SRSF1 were examined in H1299 cells with stable

overexpression of SRSF1 or control vector and stable knockdown of SRSF1 or control in a Western blot assay.

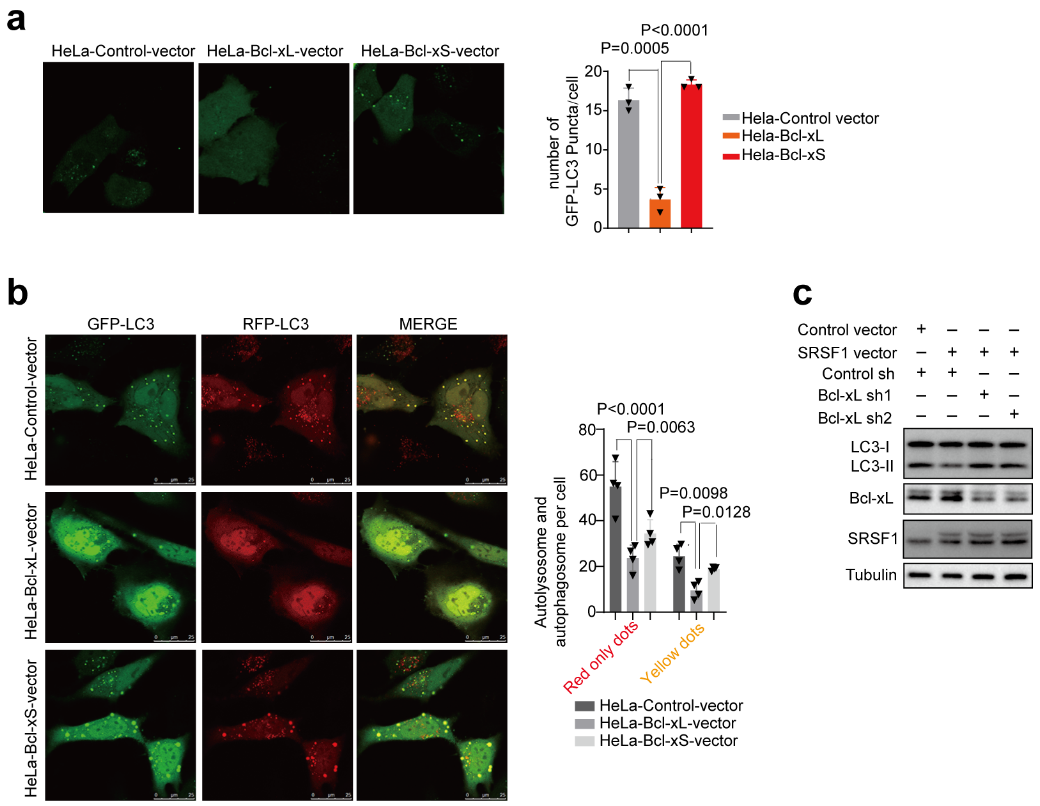

Figure. S3. The splicing of Bcl-x on autophagy regulation in HeLa cells. (a) HeLa cells with stable overexpression of Bcl-xL, Bcl-xS, or control vector were transfected with GFP-LC3. Three experiments were performed and the number of GFP-LC3 puncta per cell are represented with mean +/- SD. **(b)** HeLa cells with stably overexpressed Bcl-xL, Bcl-xS, or control vector were transfected with GFP-mRFP-LC3 to examine the expression of GFP and RFP by confocal microscope. Four experiments were carried out with mean +/– SD of the number of autophagosomes (yellow dots) and autolysosomes (red-only dots) per cell plotted. **(c)** A549 cells with stable overexpression of SRSF1 or control were stably depleted Bcl-x or control. The protein levels of LC3 and SRSF1 were examined in a Western blot assay.

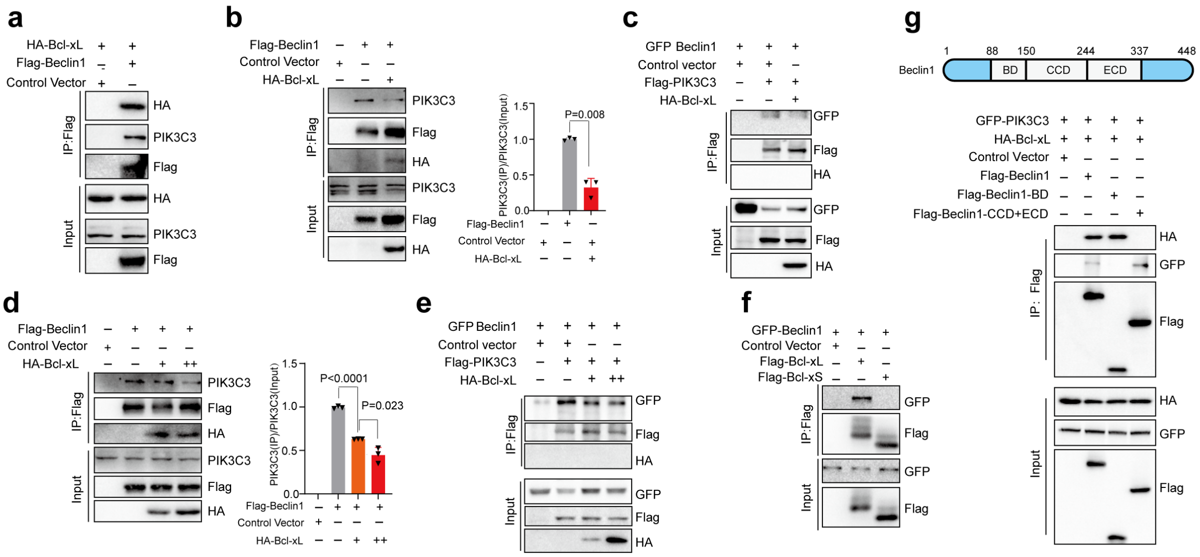

Figure. S4. SRSF1-mediated splicing of Bcl-xL inhibits autophagy through disruption of the formation of the autophagy initiation complex in H1299 cells. H1299 cells were co-transfected with different combinations of Beclin1 and Bcl-xL expression vectors in (**a-g**). Immunoprecipitation assay was carried out with anti-Flag antibody and the precipitated complexes were measured by Western blot with anti-HA, anti-Flag, or anti-PIK3C3 antibodies. (**a**) H1299 cells were co-transfected with pCDNA3-HA-Bcl-xLand pCDNA3-Flag-control, or pCDNA3-HA-Bcl-xL and pCDNA3-Flag-Beclin1 respectively. (**b**) H1299 cells were co-transfected with pCDNA3-Flag-control, or pCDNA3-Flag-Beclin1, or pCDNA3-HA-Bcl-xL and pCDNA3-Flag-Beclin1. (**c**) H1299 cells were co-transfected with pEGFP-C1-Beclin1 and pCDNA3-Flag-control, or pEGFP-C1-Beclin1 and Flag-PIK3C3 expression vector and pCDNA3-Flag-control, or pEGFP-C1-Beclin1 and Flag-PIK3C3 expression vector and pCDNA3-HA-Bcl-xL. (**d**) H1299 cells were co-transfected with pCDNA3-Flag-control, or pCDNA3-Flag-Beclin1, or pCDNA3-Flag-Beclin1 and 100 ng pCDNA3-HA-Bcl-xL, or pCDNA3-Flag-Beclin1 and 500 ng pCDNA3-HA-Bcl-xL. (**e**) H1299 cells were co-transfected with Flag-PIK3C3 and control vector; or Flag-PIK3C3, and GFP-Beclin1 expression vector; or

Flag-PIK3C3, and GFP-Beclin1 vector, with increased amounts of HA-Bcl-xL expression vectors (100ng and 500 ng) respectively. **(f)** H1299 cells were co-transfected with pEGFP-C1-Beclin1 and pCDNA3-Flag-control, or pEGFP-C1-Beclin1 and pCDNA3-Flag-Bcl-xL, or pEGFP-C1-Beclin1 and pCDNA3-Flag-Bcl-xS. (g) H1299 cells were co-transfected with pCDNA3-Flag-Beclin1, pCDNA3-HA-Bcl-xL, and pEGFP-C1-PIK3C3; or pCDNA3-Flag-Beclin1-BD, pCDNA3-HA-Bcl-xL, and pEGFP-C1-PIK3C3; or pCDNA3-Flag-Beclin1-CCD+ECD, pCDNA3-HA-Bcl-xL, and pEGFP-C1-PIK3C3.

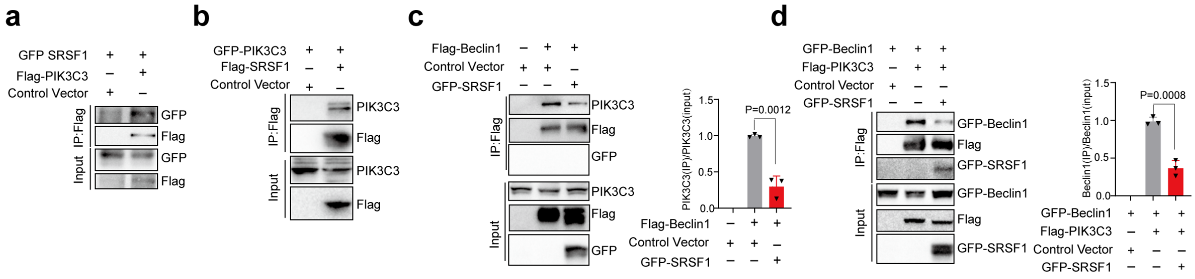

Figure. S5. SRSF1 interacts with PIK3C3 to disrupt the Beclin-PIK3C3 complex and suppresses autophagy in H1299 cells. H1299 cells were co-transfected with different combinations of Beclin1, PIK3C3, and SRSF1 expression vectors in **(a-d)**. **(a)** H1299 cells were co-transfected with pEGFP-C1-SRSF1 and pCDNA3-Flag-control; or pEGFP-C1-SRSF1 and pCDNA3-Flag-PIK3C3. **(b)** H1299 cells were co-transfected with pEGFP-C1-PIK3C3 and pCDNA3-Flag-control; or pEGFP-C1-PIK3C3 and pCDNA3-Flag-SRSF1. Immunoprecipitation assay was carried out with anti-FLAG M2 beads and the precipitated complexes were analyzed by Western blot with anti-GFP, anti-HA, anti-PIK3C3, or anti-Flag antibodies in **(a)** to **(b)**. **(c)** H1299 cells were co-transfected with pCDNA3-Flag-control; or pCDNA3-Flag-Beclin1, and pCDNA3-HA-Control; or pCDNA3-Flag-Beclin1, and pCDNA3-GFP-SRSF1. **(d)** H1299 cells were co-transfected with pEGFP-C1-Beclin1 and pCDNA3-Flag-control; or pEGFP-C1-Beclin1, and pCDNA3-Flag-PIK3C3; or pEGFP-C1-Beclin1, pCDNA3-Flag-PIK3C3 and pCDNA3-GFP-SRSF1. Immunoprecipitation assay was performed with anti-FLAG M2 beads and the precipitated complexes were analyzed by Western blot with anti-Beclin1, anti-PIK3C3, or anti-SRSF1 antibodies.

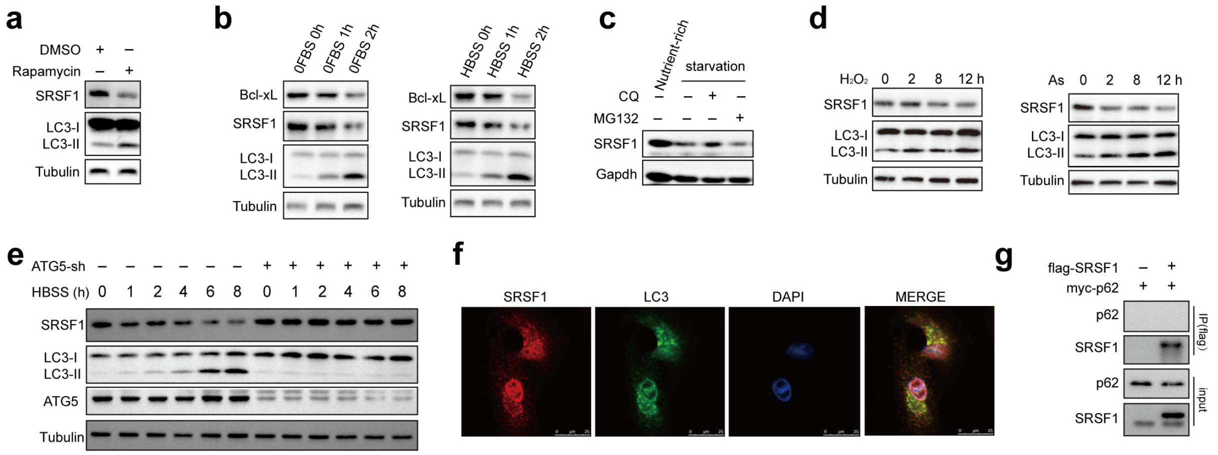

Figure. S6. SRSF1 can be degraded by oxidative stress-induced autophagy. (a) The A549 cells were treated with Rapamycin, and the treated cells were collected for a Western blot assay to examine the level of SRSF1 and LC3. **(b)** A549 cells were treated with serum free medium or HBSS medium for the indicated time. Proteins were isolated from the resulting cells and the levels of Bcl-xL, SRSF1, and LC3 were determined with a Western blot assay. **(c)** HeLa cells were treated with CQ or MG132 in the presence of serum-free medium. The protein level of SRSF1 was examined with the Western blot assay. **(d)** Autophagy was induced with hydrogen peroxide (H_2O_2) or sodium arsenite for indicated times, and the protein levels of LC3 and SRSF1 were examined. **(e)** HeLa cells with stable knockdown of ATG5 or control were treated with HBSS medium for the indicated time. The cell lysates were isolated to measure the protein levels of SRSF1, LC3-II, and ATG5 by Western blot. **(f)** The localization of SRSF1 and LC3 were examined with anti-Flag and anti-LC3 antibodies using confocal microscopy in A549 cell with stable expression of SRSF1. **(g)** 293T cells were co-transfected with pCDNA-myc-P62 and pCDNA3-flag-SRSF1 or pCDNA3-contol vectors. Co-immunoprecipitation assay was carried out with anti-FLAG antibody and the precipitated complexes were measured by Western blot with anti-P62, or anti-SRSF1antibodies.

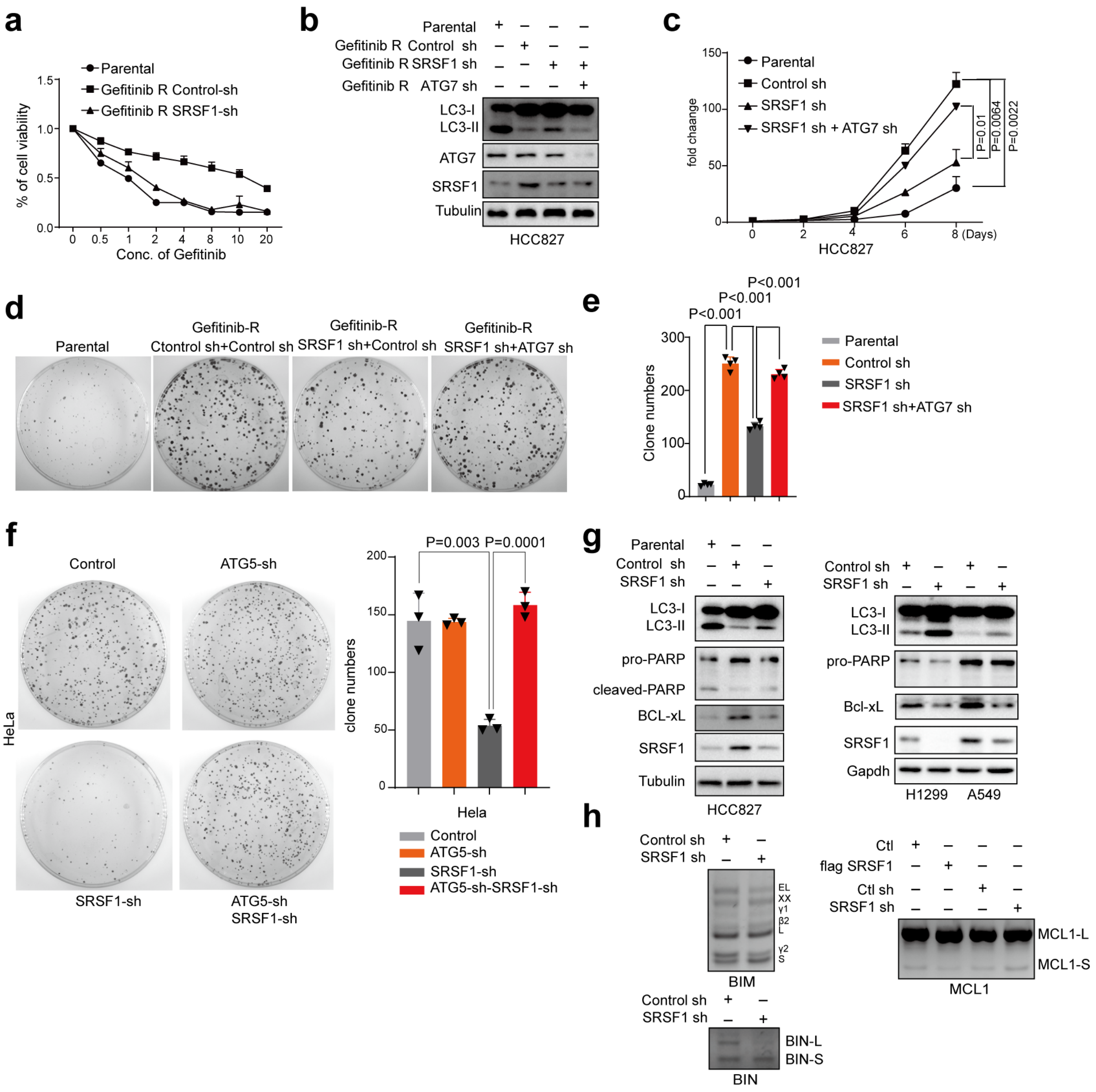

Figure. S7. Depleted ATG7 partially reversed the SRSF1 reduction-induced Gefibinib-resistant cancer cell proliferation inhibition. **(a)** The Gefitinib-resistant HCC827 cells were treated with different concentrations of Gefitinib, including 0, 0.5, 1, 2, 4, 8, 10, and 20 μM, for 24 hours. The cell viabilities of the treated cells were measured distinctly with an CCK8 assay. The percentages of cell viability were compared to concentration 0. The mean +/- SD from three experiments was plotted. **(b)** The parental cells, Gefitinib-resistant HCC827 cells with stable knockdown of control, SRSF1, SRSF1 and ATG7, were collected to determine the protein levels of LC3-II, ATG7 and SRSF1 using Western blot assay. **(c)** The parental, Gefitinib-resistant

HCC827 cells stably knocked down SRSF1, SRSF1 and ATG7, or control were grown for 8 days, with cell numbers counted every two days. The changes of cell numbers were compared to day 0. The mean +/- SD from three experiments was plotted. **(d)** The growth abilities of the parental, Gefitinib-resistant HCC827 cells with stable depletion of SRSF1, SRSF1 and ATG7, or control were determined by colony formation assay. Representative pictures of the whole plates from four experiments are shown. **(e)** The mean +/- SD of colony numbers were plotted, with p values calculated by t-test. **(f)** The growth abilities of paclitaxel-resistant HeLa cells with stable depletion of SRSF1, ATG5, SRSF1 and ATG5, or control were determined by colony formation assay. Representative pictures of the whole plates from triplicate experiments are shown. The mean +/- SD of colony numbers were plotted, with p values calculated by t-test. **(g)** The protein levels of LC3, PARP, Bcl-xL and SRSF1 were examined in parental, Gefitinib-resistant HCC827 with stable depletion of SRSF1 or control cells. The representative gels were demonstrated (left). The protein levels of LC3, PARP, Bcl-xL and SRSF1 were examined in SRSF1 or control stably knocked down or overexpressed H1299 and A549 cells. The representative gels were demonstrated (right). (**h**) The splicing of BIM, BIN, and MCL1 was measured in SRSF1 stably depleted A549 cells using RT-PCR. The representative gels were shown.
